# Assessing the real-world safety of tralokinumab for atopic dermatitis: insights from a comprehensive analysis of FAERS data

**DOI:** 10.3389/fphar.2024.1458438

**Published:** 2024-08-13

**Authors:** Kaidi Zhao, Yang Zhao, Shengxiang Xiao, Chen Tu

**Affiliations:** ^1^ Department of Dermatology, Second Affiliated Hospital of Xi’an Jiaotong University, Xi’an, China; ^2^ National & Local Joint Engineering Research Center of Biodiagnosis and Biotherapy, The Second Affiliated Hospital of Xi’an Jiaotong University, Xi’an, China

**Keywords:** tralokinumab, atopic dermatitis, FAERS, disproportionality analysis, conjunctivitis

## Abstract

**Background:**

Tralokinumab, a humanized monoclonal antibody targeting interleukin-13, has been primarily used for the treatment of moderate-to-severe atopic dermatitis. Given its extensive use in clinical practice, understanding its safety profile in the real-world setting is crucial.

**Methods:**

This study utilized disproportionality analysis to evaluate the safety of tralokinumab in clinical practice by analyzing all adverse event reports since 2021 in the FDA Adverse Event Reporting System database that identified tralokinumab as the primary suspected drug. Reporting odds ratio, proportional reporting ratio, multi-item gamma Poisson shrinker, and Bayesian confidence propagation neural network were used for disproportionality analyses of adverse events related to tralokinumab. Additionally, the Weibull distribution was employed to model the risk of adverse events over time.

**Results:**

Adverse reactions documented on the drug label, such as injection site reactions, conjunctivitis, and upper respiratory infections, displayed positive signals. Additionally, potential adverse reactions not mentioned on the label were also identified, including dizziness, headache, nausea, vomiting, hair loss, and acne. The importance of adverse event monitoring, particularly in the first month after treatment initiation, was emphasized.

**Conclusion:**

This study has provided preliminary safety data on the real-world application of tralokinumab, confirming some known adverse reactions and revealing additional potential risks. The findings offer critical safety information for clinicians prescribing tralokinumab to treat atopic dermatitis.

## 1 Introduction

Tralokinumab, a humanized monoclonal antibody targeting interleukin-13 (IL-13), represents a significant advancement in treating moderate-to-severe atopic dermatitis (Müller et al., 2024). With the increasing global prevalence of atopic dermatitis, this condition significantly impairs patients’ quality of life and places substantial burdens on healthcare systems (Langan et al., 2020). The introduction of tralokinumab permitted a targeted therapeutic approach that aligns with the pathophysiological mechanisms of atopic dermatitis, marking a new era in managing this chronic skin disease (Popovic et al., 2017; Wollenberg et al., 2019; Zhang et al., 2022).

Tralokinumab exerts its therapeutic effects by neutralizing the activity of IL-13, a crucial cytokine in the inflammatory cascade of atopic dermatitis (Popovic et al., 2017; Zhang et al., 2022). Its efficacy has been validated in numerous Phase II and III clinical trials, demonstrating significant improvements in the eczema area and severity index, investigator’s global assessment score, dermatology life quality index, and symptoms such as itching and sleep disruption (Wollenberg et al., 2019; Wollenberg et al., 2021; Gutermuth et al., 2022). Although the short-term safety profile of tralokinumab has been established through multiple studies (Silverberg et al., 2021; Gutermuth et al., 2022), a comprehensive evaluation of its long-term safety in clinical practice is lacking.

The FDA Adverse Event Reporting System (FAERS) compiles drug adverse event reports voluntarily submitted by consumers, physicians, and pharmacists. It serves as an essential resource for assessing the real-world safety of medications (Shu et al., 2022a; Shu et al., 2022b). Recently, FAERS was used to identify adverse events associated with various drugs, such as antiviral drug-associated DRESS syndrome (Sharma and Kumar, 2022; Sharma et al., 2023) and the effects of proton pump inhibitors on the renal system (Jain et al., 2023).

This study assessed the safety profile of tralokinumab in real-world settings by analyzing data from the FAERS database through disproportionality analysis. The findings could provide clinicians with guidance on the safe administration of this medication.

## 2 Materials and methods

### 2.1 Data sources, management, and study design

**FIGURE 1:**
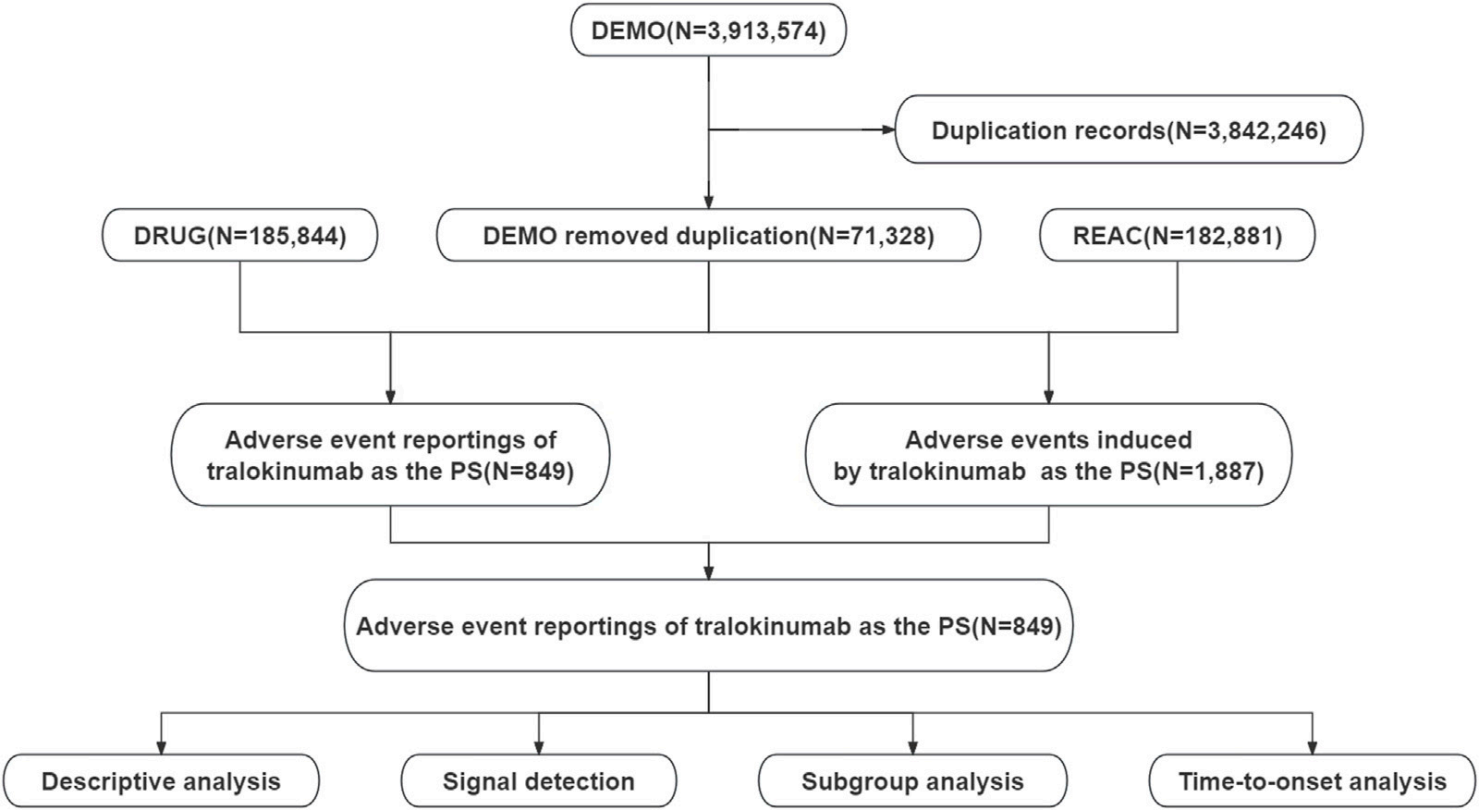
Flowchart demonstrating the adverse event analysis process for tralokinumab using the FDA Adverse Event Reporting System database.

This study used data from the publicly accessible FAERS database, a spontaneous reporting system in which reports are primarily submitted by consumers, physicians, and pharmacists. The analysis included all adverse event reports in which tralokinumab was identified as the primary suspected drug, and the search period spanned from the fourth quarter of 2021 to the fourth quarter of 2023. The data management process involved removing duplicate reports and standardizing adverse event terminology. Duplicates were processed according to FDA-recommended practices. Specifically, for reports with identical case identifiers (CASEIDs), reports with the highest FDA receipt date (FDA_DT) were retained. When the CASEID and FDA_DT values matched, the report with the highest PRIMARYID (the unique identifier assigned to each report) was retained. Adverse event terms were standardized using the MedDRA dictionary, version 26.1, thereby enhancing the reliability of subsequent statistical analyses. Figure 1 presents a detailed flowchart of the study design.

### 2.2 Statistical analysis

Descriptive analysis was employed to characterize the features of adverse event reports associated with tralokinumab. Four disproportionality analysis methods were used to detect signals of potential adverse reactions attributable to tralokinumab: reporting odds ratio (ROR) (Rothman et al., 2004), proportional reporting ratio (PRR) (Evans et al., 2001), multi-item gamma Poisson shrinker (MGPS) (Rivkees and Szarfman, 2010), and Bayesian confidence propagation neural network (BCPNN) (Ang et al., 2016). An adverse event meeting the positivity threshold for at least one method was considered a potential adverse reaction. Detailed two-by-two contingency tables are provided in Supplementary Table S1. The formulas and thresholds for these disproportionality analyses are outlined in Supplementary Table S2. The interval between the occurrence of adverse events (as reported in the DEMO file) and the initiation of tralokinumab treatment (as recorded in the THER file) defined the onset time of tralokinumab-related adverse events. The Weibull distribution was applied to model changes in the incidence of adverse events over time. All analyses were performed using R software version 4.2.2.

## 3 Results

### 3.1 Descriptive analysis

This investigation encompassed 849 reports of adverse events (1887 total adverse events) in which tralokinumab was the suspected primary agent. Of these, 60.5% involved female patients, whereas 38.5% involved male patients. The predominant age group was 18–65 years, accounting for 40.3% of the cases. Most reports (65%) were submitted by healthcare professionals, with 92.3% originating from the United States, followed by 1.9% from Germany and 1.6% from Canada. Further descriptive outcomes are presented in Table 1.

**TABLE 1:** Clinical characteristics of adverse event reports related to tralokinumab from the FDA Adverse Event Reporting System database (Q4 2021–Q4 2023).

Characteristics	Number of cases	Proportion of cases (%)
Number of events	849	
Sex		
Male	327	38.5
Female	514	60.5
Missing	8	0.9
Age		
Median (interquartile range)	57 (41, 66)	
<18	2	0.2
18–65	342	40.3
65–85	122	14.4
>85	6	0.7
Missing	377	44.4
Top 5 reporting countries		
United States	784	92.3
Germany	16	1.9
Canada	14	1.6
France	12	1.4
Spain	5	0.6
Reporter		
Healthcare professional	552	65.0
Non-healthcare professional	240	28.3
Missing	57	6.7
Reporting year		
2022	235	27.7
2023	614	72.3

### 3.2 Distribution of adverse events at the system organ class (SOC) level

**FIGURE 2:**
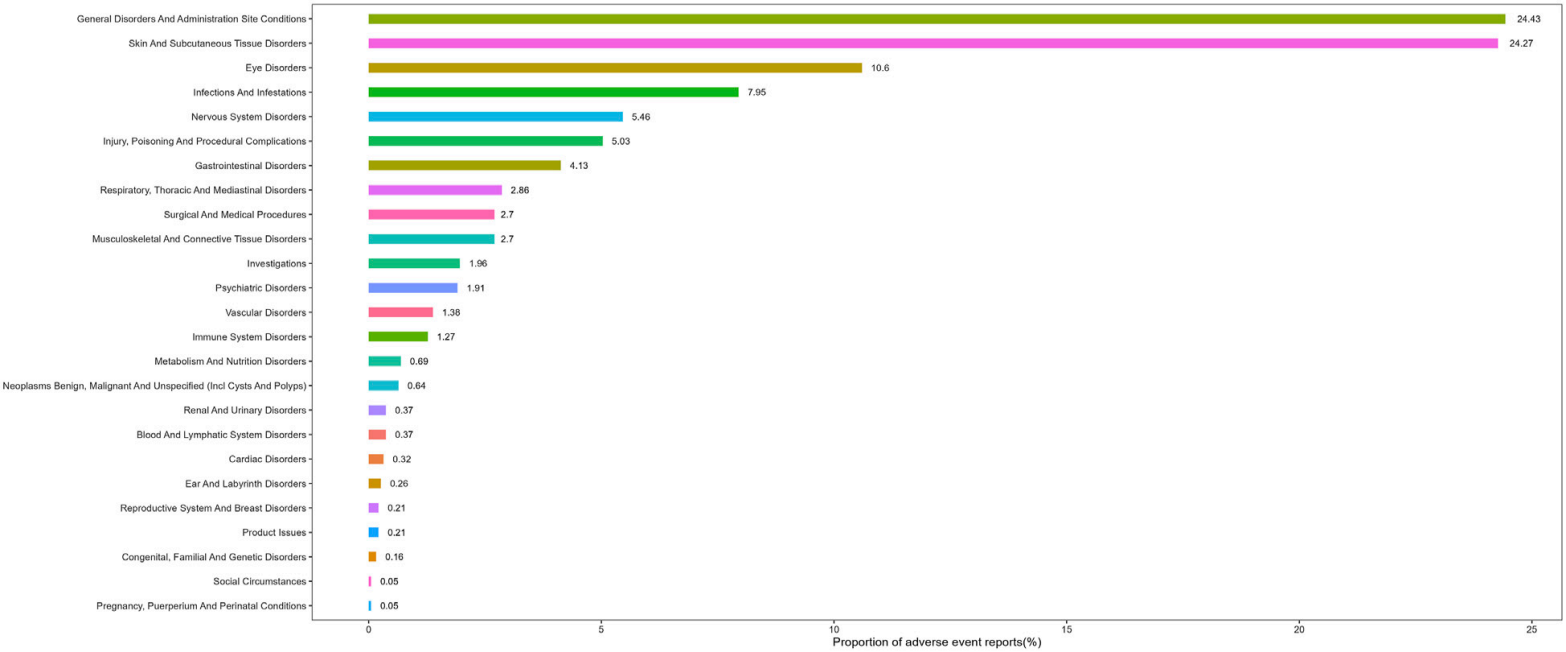
Proportion of adverse events by system organ class for tralokinumab.

Adverse events associated with tralokinumab encompassed 25 of the 27 SOCs. As presented in Table 2, significant findings were observed in several categories, including general disorders and administration site conditions, gastrointestinal disorders, nervous system disorders, infections and infestations, eye disorders, vascular disorders, surgical and medical procedures, and congenital, familial, and genetic disorders. The distribution of adverse events at the SOC level is depicted in Figure 2.

**TABLE 2:** Signal strength of tralokinumab-related adverse events across system organ classes (SOCs) in the FDA Adverse Event Reporting System database.

SOC	Number of cases	ROR (95% CI)	PRR (χ2)	EBGM (EBGM05)	IC (IC025)
Skin and subcutaneous tissue disorders	458	0.78 (0.7–0.87)	0.83 (21.31)	0.83 (0.76)	−0.26 (−1.93)
General disorders and administration site conditions*	461	1.34 (1.2–1.49)	1.25 (29.19)	1.25 (1.15)	0.32 (−1.34)
Gastrointestinal disorders*	78	1.55 (1.23–1.95)	1.53 (14.28)	1.52 (1.25)	0.6 (−1.07)
Respiratory, thoracic, and mediastinal disorders	54	1.15 (0.88–1.51)	1.15 (1.01)	1.14 (0.91)	0.19 (−1.47)
Neoplasms benign, malignant, and unspecified (including cysts and polyps)	12	1.19 (0.67–2.1)	1.19 (0.35)	1.19 (0.74)	0.25 (−1.42)
Nervous system disorders*	103	1.78 (1.45–2.17)	1.74 (32.54)	1.72 (1.46)	0.78 (−0.88)
Musculoskeletal and connective tissue disorders	51	0.79 (0.6–1.04)	0.79 (2.84)	0.79 (0.63)	−0.33 (−2)
Infections and infestations*	150	1.41 (1.2–1.67)	1.38 (16.52)	1.38 (1.2)	0.46 (−1.21)
Eye disorders*	200	1.33 (1.15–1.55)	1.3 (14.74)	1.29 (1.14)	0.37 (−1.29)
Blood and lymphatic system disorders	7	1.22 (0.58–2.58)	1.22 (0.28)	1.22 (0.65)	0.28 (−1.39)
Vascular disorders*	26	2.47 (1.67–3.65)	2.44 (21.77)	2.41 (1.74)	1.27 (−0.4)
Injury, poisoning, and procedural complications	95	0.32 (0.26–0.39)	0.36 (128.83)	0.36 (0.3)	−1.48 (−3.15)
Psychiatric disorders	36	0.53 (0.38–0.73)	0.54 (14.91)	0.54 (0.41)	−0.89 (−2.56)
Surgical and medical procedures*	51	2 (1.51–2.65)	1.98 (24.44)	1.96 (1.55)	0.97 (−0.7)
Cardiac disorders	6	0.69 (0.31–1.53)	0.69 (0.85)	0.69 (0.35)	−0.54 (−2.21)
Investigations	37	1.19 (0.85–1.65)	1.18 (1.04)	1.18 (0.9)	0.24 (−1.43)
Immune system disorders	24	1.5 (1–2.25)	1.49 (3.88)	1.49 (1.06)	0.57 (−1.1)
Metabolism and nutrition disorders	13	1.62 (0.93–2.81)	1.62 (3.01)	1.6 (1.01)	0.68 (−0.99)
Pregnancy, puerperium, and perinatal conditions	1	0.98 (0.14–7.02)	0.98 (0)	0.98 (0.19)	−0.03 (−1.72)
Congenital, familial, and genetic disorders*	3	5.14 (1.61–16.45)	5.14 (9.49)	4.93 (1.86)	2.3 (0.59)
Product issues	4	0.66 (0.25–1.77)	0.66 (0.69)	0.66 (0.29)	−0.59 (−2.26)
Renal and urinary disorders	7	1.21 (0.57–2.55)	1.21 (0.25)	1.2 (0.65)	0.27 (−1.4)
Reproductive system and breast disorders	4	0.84 (0.31–2.25)	0.84 (0.12)	0.84 (0.37)	−0.25 (−1.92)
Ear and labyrinth disorders	5	1.19 (0.49–2.87)	1.19 (0.15)	1.18 (0.57)	0.24 (−1.43)
Social circumstances	1	0.12 (0.02–0.83)	0.12 (6.66)	0.12 (0.02)	−3.08 (−4.75)

Asterisks (*) indicate statistically significant signals. Abbreviations: ROR, reporting odds ratio; PRR, proportional reporting ratio; EBGM, empirical Bayesian geometric mean; EBGM05, the lower limit of the 95% confidence interval of EBGM; IC, information component; IC025, the lower limit of the 95% confidence interval of the IC.

### 3.3 Distribution of adverse events at the preferred term (PT) level

Adverse events associated with tralokinumab were ranked by frequency and evaluated for positive signals. Among the 50 most common adverse events, known reactions including injection site erythema, injection site pruritus, conjunctivitis, hypersensitivity, and nasopharyngitis were confirmed. Furthermore, potential adverse reactions not listed on the label, such as headache, fatigue, dizziness, urticaria, nausea, vomiting, hair loss, syncope, and urinary tract infections, were additionally identified. Detailed information on these findings is presented in Table 3. All adverse events that met the criteria for a positive signal are documented in Supplementary Table S3.

**TABLE 3:** Top 50 most frequent adverse events for tralokinumab at the preferred term (PT) level.

PT	Number of cases	ROR (95% CI)	PRR (χ2)	EBGM (EBGM05)	IC (IC025)
Pruritus	107	1.14 (0.94–1.39)	1.14 (1.79)	1.13 (0.96)	0.18 (−1.49)
Drug ineffective*	97	1.67 (1.36–2.06)	1.64 (24.49)	1.63 (1.37)	0.7 (−0.96)
Dermatitis atopic	44	0.44 (0.33–0.6)	0.46 (29.98)	0.46 (0.36)	−1.13 (−2.79)
Rash	44	0.57 (0.42–0.76)	0.58 (14.29)	0.58 (0.45)	−0.79 (−2.46)
Injection site pain	41	0.97 (0.71–1.33)	0.97 (0.03)	0.97 (0.75)	−0.04 (−1.71)
Erythema*	37	1.65 (1.19–2.29)	1.63 (9.06)	1.62 (1.23)	0.7 (−0.97)
Dry skin	34	0.76 (0.54–1.07)	0.77 (2.42)	0.77 (0.58)	−0.38 (−2.05)
Headache*	32	2.69 (1.89–3.83)	2.66 (32.52)	2.62 (1.95)	1.39 (−0.28)
Incorrectly dose administered*	32	1.69 (1.19–2.41)	1.68 (8.8)	1.67 (1.24)	0.74 (−0.93)
Fatigue*	29	2.7 (1.86–3.91)	2.67 (29.69)	2.63 (1.92)	1.39 (−0.28)
Injection site erythema*	28	2.24 (1.54–3.27)	2.22 (18.5)	2.19 (1.6)	1.13 (−0.54)
Conjunctivitis*	27	2.38 (1.62–3.5)	2.37 (20.89)	2.33 (1.69)	1.22 (−0.45)
Dizziness*	25	3.58 (2.4–5.35)	3.55 (44.27)	3.46 (2.47)	1.79 (0.12)
Eye pruritus	25	1.4 (0.94–2.08)	1.39 (2.77)	1.39 (1)	0.47 (−1.2)
Skin exfoliation	23	0.82 (0.54–1.24)	0.82 (0.91)	0.82 (0.58)	−0.28 (−1.95)
Dry eye	23	1.12 (0.74–1.69)	1.12 (0.28)	1.11 (0.79)	0.16 (−1.51)
Eczema	23	0.59 (0.39–0.89)	0.59 (6.56)	0.59 (0.42)	−0.75 (−2.42)
Injection site pruritus*	22	2.85 (1.86–4.36)	2.82 (25.3)	2.77 (1.94)	1.47 (−0.2)
Arthralgia	22	0.94 (0.62–1.44)	0.95 (0.07)	0.95 (0.66)	−0.08 (−1.75)
Urticaria*	21	2.35 (1.52–3.63)	2.33 (15.66)	2.3 (1.6)	1.2 (−0.47)
Ocular hyperemia	20	1.26 (0.81–1.96)	1.26 (1.05)	1.25 (0.87)	0.33 (−1.34)
Injection site swelling	19	1.27 (0.81–2)	1.27 (1.08)	1.27 (0.86)	0.34 (−1.33)
Condition aggravated	19	0.93 (0.59–1.46)	0.93 (0.1)	0.93 (0.64)	−0.1 (−1.77)
Nausea*	18	2.54 (1.58–4.06)	2.52 (16.15)	2.48 (1.67)	1.31 (−0.36)
Therapy interrupted*	16	8.45 (5.06–14.11)	8.39 (95.82)	7.79 (5.07)	2.96 (1.28)
Malaise*	15	2.79 (1.67–4.67)	2.78 (16.63)	2.73 (1.77)	1.45 (−0.22)
Hypersensitivity*	15	2.11 (1.26–3.52)	2.1 (8.46)	2.07 (1.35)	1.05 (−0.62)
Alopecia*	15	2.28 (1.37–3.82)	2.27 (10.48)	2.24 (1.46)	1.17 (−0.51)
Injection site rash*	15	2.81 (1.68–4.71)	2.8 (16.9)	2.75 (1.79)	1.46 (−0.21)
Eye pain*	15	2.95 (1.76–4.94)	2.94 (18.63)	2.88 (1.87)	1.53 (−0.15)
Nasopharyngitis*	14	1.84 (1.08–3.13)	1.83 (5.21)	1.82 (1.17)	0.86 (−0.81)
Vision blurred	14	1.13 (0.67–1.92)	1.13 (0.22)	1.13 (0.73)	0.18 (−1.49)
Pain	13	0.9 (0.52–1.56)	0.9 (0.15)	0.9 (0.57)	−0.15 (−1.82)
Injection site reaction	13	1.57 (0.9–2.71)	1.56 (2.6)	1.55 (0.98)	0.64 (−1.03)
Syncope*	12	10.52 (5.79–19.13)	10.46 (92.67)	9.53 (5.78)	3.25 (1.57)
COVID-19	12	0.62 (0.35–1.1)	0.63 (2.7)	0.63 (0.39)	−0.67 (−2.34)
Skin fissures	11	0.81 (0.45–1.47)	0.81 (0.48)	0.81 (0.49)	−0.3 (−1.97)
Eye irritation	11	0.69 (0.38–1.25)	0.69 (1.5)	0.7 (0.42)	−0.52 (−2.19)
Cough	11	1.45 (0.8–2.64)	1.45 (1.52)	1.44 (0.88)	0.53 (−1.14)
Dyspnea	11	1.64 (0.9–2.98)	1.64 (2.68)	1.63 (0.99)	0.7 (−0.97)
Injection site bruising	10	1.54 (0.83–2.89)	1.54 (1.88)	1.53 (0.91)	0.62 (−1.05)
Lacrimation increased	9	1.35 (0.7–2.61)	1.35 (0.8)	1.34 (0.77)	0.43 (−1.24)
Visual impairment	9	1.26 (0.65–2.44)	1.26 (0.49)	1.26 (0.73)	0.33 (−1.34)
Diarrhea	9	1.62 (0.84–3.13)	1.61 (2.07)	1.6 (0.92)	0.68 (−0.99)
Illness	9	1.09 (0.57–2.11)	1.09 (0.07)	1.09 (0.63)	0.12 (−1.55)
Inappropriate schedule of product administration	9	0.35 (0.18–0.67)	0.35 (10.79)	0.36 (0.21)	−1.49 (−3.16)
Skin burning sensation	8	1.26 (0.63–2.54)	1.26 (0.43)	1.26 (0.7)	0.33 (−1.34)
Vomiting*	8	2.23 (1.1–4.5)	2.22 (5.28)	2.2 (1.22)	1.14 (−0.54)
Sleep disorder	8	0.29 (0.15–0.59)	0.3 (13.54)	0.3 (0.17)	−1.75 (−3.41)
Urinary tract infection*	8	2.22 (1.1–4.49)	2.22 (5.24)	2.19 (1.22)	1.13 (−0.54)

Asterisks (*) indicate statistically significant signals. Abbreviations: ROR, reporting odds ratio; PRR, proportional reporting ratio; EBGM, empirical Bayesian geometric mean; EBGM05, the lower limit of the 95% confidence interval of EBGM; IC, information component; IC025, the lower limit of the 95% confidence interval of the IC.

### 3.4 Subgroup analysis

Subgroup analysis of adverse events associated with tralokinumab revealed that among the 50 most common adverse events meeting the positive signal criteria, events occurring exclusively in males included diarrhea and vomiting, whereas acne and muscle spasms were specific to females. Specific details are presented in Supplementary Tables S4, S5. Only two adverse event reports involved patients younger than 18 years, and they did not feature events beyond those specified on the drug label. In patients aged 18–65 years, the 50 most frequent adverse events displaying positive signals included headache, dizziness, syncope, fatigue, nausea, vomiting, diarrhea, hair loss, and urticaria. For patients older than 65 years, common adverse events included fatigue, urinary tract infection, and facial swelling (Supplementary Tables S6–S8).

### 3.5 Sensitivity analysis

Tralokinumab is commonly used in combination with medications such as prednisone, budesonide, clobetasol, tacrolimus, and pimecrolimus. After excluding reports involving the concurrent use of other drugs, we identified 806 reports involving 1733 adverse events. Persistent potential adverse reactions included headache, fatigue, dizziness, hives, nausea, hair loss, fainting, and urinary tract infections (Supplementary Table S9).

### 3.6 Time to onset and weibull distribution analysis of adverse events

**FIGURE 3:**
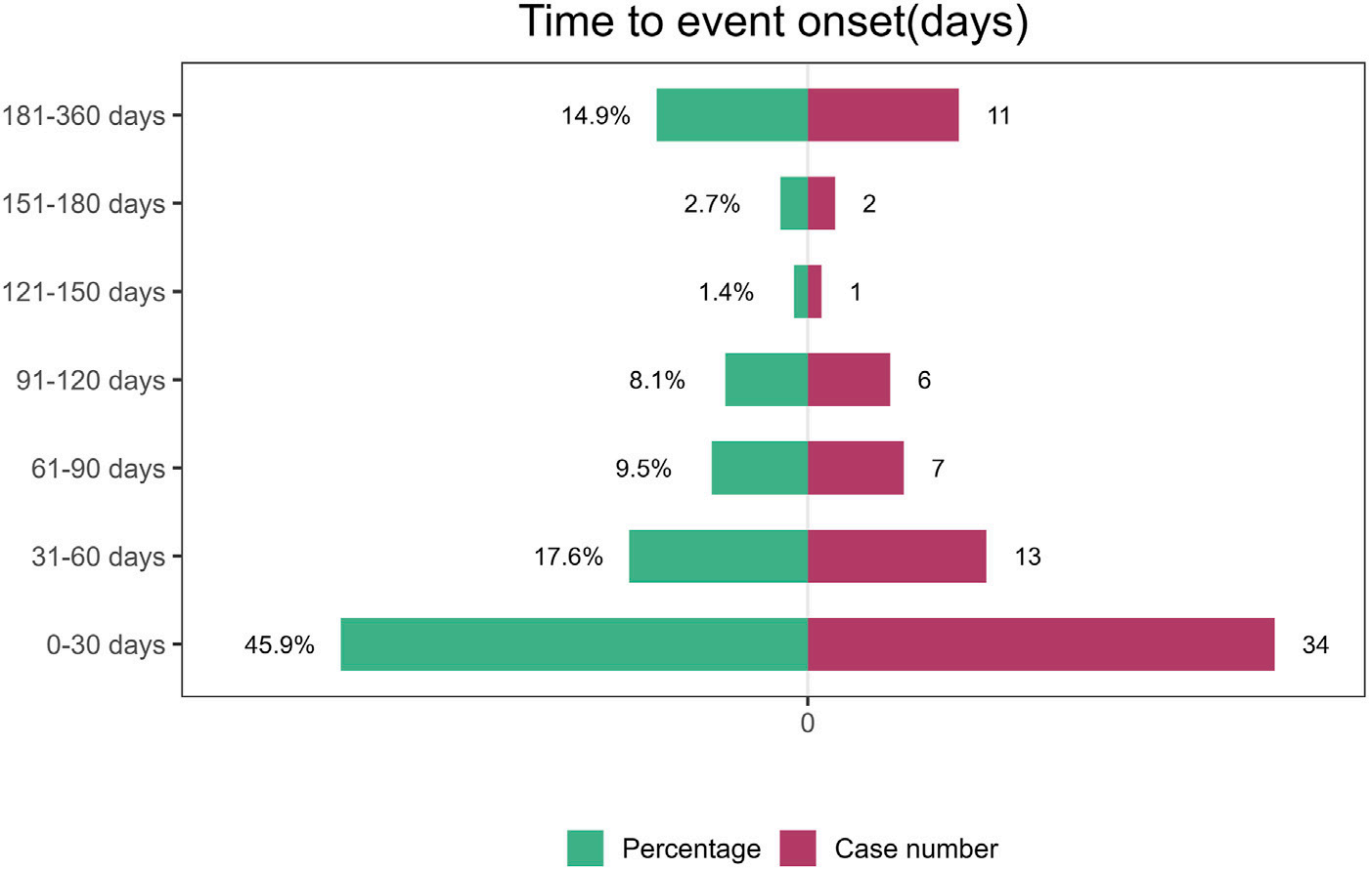
Time to onset of adverse events induced by tralokinumab.

**FIGURE 4:**
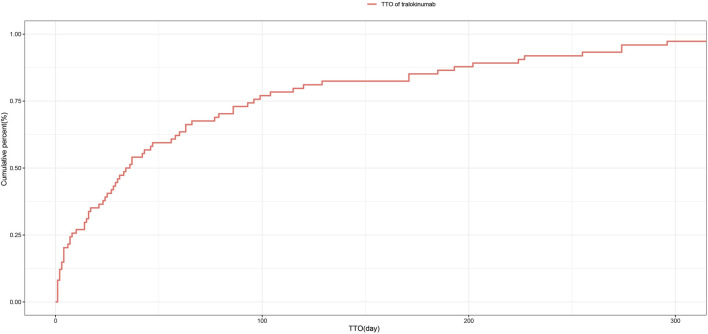
Cumulative incidence of adverse events related to tralokinumab over time.

Regarding the time to onset, adverse events associated with tralokinumab primarily occurred within the first month of treatment. The specific temporal distribution of these events is depicted in Figure 3. Additionally, the cumulative incidence curve of adverse events is illustrated in Figure 4. Analysis using the Weibull distribution indicated an early failure mode. The detailed parameters of this analysis are provided in Table 4.

**TABLE 4:** Time to onset of tralokinumab-associated adverse events and Weibull distribution analysis.

Drug	Time to onset (days)		Weibull distribution		
	Case reports	Median (IQR)	Scale parameter: α (95% CI)	Shape parameter: β (95%CI)	Type
Tralokinumab	74	35 (8.50–95.25)	59.94 (40.35–79.52)	0.74 (0.60–0.87)	Early failure

Abbreviations: CI, confidence interval; IQR, interquartile range.

## 4 Discussion

This study comprehensively assessed adverse events associated with tralokinumab following its market launch in 2021. By analyzing data from FAERS, this research confirmed previously identified adverse reactions included on the tralokinumab drug label, including injection site reactions, conjunctivitis, hypersensitivity, upper respiratory infections, and nasopharyngitis. Additionally, adverse events not cited on the label, including neurological symptoms (e.g., dizziness, headache, syncope), gastrointestinal disturbances (e.g., nausea, vomiting), and dermatological reactions (e.g., urticaria, hair loss), were also uncovered. These findings emphasize the need for drug monitoring, particularly within the first month of treatment initiation, to effectively manage and mitigate potential adverse effects.

Multiple clinical trials describe conjunctivitis as a common adverse reaction of tralokinumab. For instance, a Phase II trial of tralokinumab in the treatment of atopic dermatitis reported conjunctivitis rates of 5.9% at a dosage of 150 mg and 2% at a dosage of 45 mg (Wollenberg et al., 2019). Further, an analysis of five clinical trials involving 2,285 adults with moderate-to-severe atopic dermatitis detected a higher incidence of conjunctivitis in the tralokinumab group (7.5%) than in the placebo group (3.2%) (Wollenberg et al., 2022). Our findings are consistent with previous research. It is crucial to monitor and manage this adverse reaction vigilantly when administering tralokinumab for atopic dermatitis.

Another adverse reaction warranting attention is upper respiratory infection. Consistent with our results, the incidence of upper respiratory infections was higher for tralokinumab than for placebo in both 16- and 52-week clinical trials (Silverberg et al., 2021; Wollenberg et al., 2021). This adverse reaction has also been observed in patients who received tralokinumab for asthma. Although tralokinumab effectively reduces airway inflammation and mucus secretion in asthma, potentially alleviating symptoms (Brightling et al., 2015), it can also induce treatment-emergent adverse events such as upper respiratory infections (Busse et al., 2019). Upper respiratory infections can decrease medication adherence among patients. Therefore, when using tralokinumab to treat atopic dermatitis, clinicians should be vigilant regarding the possibility of upper respiratory infections.

Additionally, our study revealed several adverse events not currently listed on the drug label. Notably, tralokinumab can induce neurological reactions, including headache and dizziness. The precise mechanism by which tralokinumab triggers headache remains to be elucidated. Although not documented as an adverse reaction on the drug label, headache was frequently reported in multiple clinical trials of tralokinumab for treating atopic dermatitis (Wollenberg et al., 2021). Moreover, dizziness could be a manifestation of underlying vascular conditions. For instance, in a case report of dupilumab, a monoclonal antibody targeting IL-4Rα used to treat atopic dermatitis, the patient developed ischemic stroke characterized by dizziness and nausea. This condition was possibly exacerbated by the inhibition of IL-4 and IL-13 by dupilumab, resulting in the activation of the pro-inflammatory cytokines IL-1β and TNF-α in endothelial cells, thereby activating coagulation pathways and promoting thrombus formation (Iwase et al., 2020). Similarly, tralokinumab’s specific inhibition of IL-13 might precipitate adverse events akin to those observed with dupilumab, though the exact pathways for tralokinumab-induced headache and dizziness warrant further investigation. These neurological adverse events can profoundly affect patients’ quality of life, especially among individuals susceptible to atopic dermatitis (Lugović-Mihić et al., 2023). Therefore, these unlisted adverse events should be closely monitored and seriously considered by clinicians.

Nausea and vomiting are potential adverse reactions not mentioned on the drug label that could limit the clinical use of tralokinumab. These gastrointestinal symptoms can cause substantial physiological and psychological distress, potentially leading to treatment discontinuation. It is therefore imperative to meticulously monitor patients’ responses following tralokinumab administration. Timely intervention with antiemetic medications such as ondansetron can effectively control these symptoms and enhance patient adherence to the treatment regimen.

Additionally, this study identified hair loss as a potential adverse reaction of tralokinumab. Notably, atopic dermatitis affects both children and a significant proportion of adults. In high-income countries, the incidence of atopic dermatitis among adults is approximately 10%, and its prevalence is increasing globally (Langan et al., 2020). Research indicates that hair loss in adults can have extensive psychological and emotional repercussions extending beyond physical symptoms (Gorbatenko-Roth et al., 2023; Muntyanu et al., 2023; Clemmesen et al., 2024). Hair loss can lead to anxiety, depression, social withdrawal, and reduced self-esteem, which can severely affect patients’ overall quality of life and work productivity (Gandhi et al., 2023). Given that hair loss represents a potential adverse reaction not listed on the drug label, it is crucial for patients receiving tralokinumab treatment for atopic dermatitis to be preemptively informed about the risk of hair loss. Proactive management of this and other adverse reactions is essential to enhance the overall quality of life of patients.

The subgroup analysis emphasized that gastrointestinal symptoms, such as diarrhea and vomiting, should receive additional attention in males, whereas vigilance regarding the risks of acne and muscle spasms is needed in females. Notably, acne can cause significant psychological stress, especially in females, potentially leading to anxiety, depression, and even suicidal thoughts (Altunay et al., 2020; Hughes and Bewley, 2023). In the pediatric population, no adverse reactions were observed beyond those described in the drug label. In elderly patients older than 65, vigilance is warranted concerning urinary tract infections, which can adversely affect their quality of life. Through sensitivity analysis, we identified persistent potential adverse reactions associated with tralokinumab monotherapy, including headache and fatigue. Such non-lethal but impactful adverse events can influence treatment adherence, adversely affecting therapeutic efficacy. Given these findings, it is crucial to pay careful attention to these specific adverse events to optimize treatment outcomes and enhance medication effectiveness.

This study additionally conducted a temporal analysis of adverse events and employed the Weibull distribution to predict the timing of these events, facilitating the establishment of effective timelines for monitoring drug-related adverse reactions. The results underscore the critical importance of vigilant monitoring, particularly during the first month of tralokinumab treatment. This early monitoring period is crucial for detecting and managing potential adverse reactions, thereby optimizing patient safety and treatment outcomes.

This study had several limitations. First, the FAERS database, as a spontaneous reporting system utilized by consumers, physicians, and pharmacists, can inherently contain missing or inaccurate data. For example, data related to patient exposure to medicines are not available. Second, the volume of data analyzed in this study was limited, and larger datasets are necessary to validate our findings. However, our study both focused on the medication itself and specifically addressed its indications for use, thereby enhancing the specificity of the results. Third, the majority of the data were sourced from the United States, which might have introduced reporting bias. Future research should aim to incorporate data from multiple countries to enhance generalizability. Lastly, although disproportionality analysis effectively identified positive signals of adverse events, it did not establish a causal relationship between tralokinumab and these events. Long-term prospective studies are necessary to confirm the potential adverse reactions identified in this research.

## 5 Conclusion

In this study, we conducted a comprehensive analysis of adverse events associated with tralokinumab using the FAERS database, focusing on reports published since 2021. The analysis both confirmed known adverse reactions and identified potential adverse reactions not mentioned on the label, such as dizziness, headache, nausea, vomiting, hair loss, and acne. These findings offer essential safety information for clinicians prescribing tralokinumab and underscore the necessity to closely monitor patients for potential adverse reactions during the treatment of atopic dermatitis.

## Data Availability

The datasets presented in this study can be found in online repositories. The names of the repository/repositories and accession number(s) can be found below: https://fis.fda.gov/extensions/FPD-QDE-FAERS/FPD-QDE-FAERS.html.
