# Supplementary material for: Assessing the real-world safety of tralokinumab for atopic dermatitis: insights from a comprehensive analysis of FAERS data
Source: Front Pharmacol. 2024 Aug 13;15:1458438. doi: 10.3389/fphar.2024.1458438 (PMC11347326; doi:10.3389/fphar.2024.1458438)
Supplement: Supplementary file 1 [file Table1.docx]

Supplementary Material

Supplementary Tables

Supplementary Table 1:

Two-by-two contingency table for disproportionality analyses.

|  | Target AEs | Other AEs | Total |
| --- | --- | --- | --- |
| Tralokinumab | a | b | a+b |
| Other drugs | c | d | c+d |
| Total | a+c | b+d | a+b+c+d |

Abbreviation: AEs, adverse events; a, number of reports containing both the target drug and target adverse drug reaction; b, number of reports containing other adverse drug reaction of the target drug; c, number of reports containing the target adverse drug reaction of other drugs; d, number of reports containing other drugs and other adverse drug reactions.

Supplementary Table 2:

Four major algorithms used for signal detection.

| Algorithms | Equation | Criteria |
| --- | --- | --- |
| ROR | ROR=ad/b/c | lower limit of 95% CI>1, N≥3 |
|  | 95%CI=e^ln(ROR)±1.96(1/a+1/b+1/c+1/d)^0.5^ |  |
| PRR | PRR=a(c+d)/c/(a+b) | PRR≥2, χ^2^≥4, N≥3 |
|  | χ^2^=[(ad-bc)^2](a+b+c+d)/[(a+b)(c+d)(a+c)(b+d)] |  |
| BCPNN | IC=log_2_a(a+b+c+d)(a+c)(a+b) | IC025>0 |
|  | 95%CI= E(IC) ± 2V(IC)^0.5 |  |
| MGPS | EBGM=a(a+b+c+d)/(a+c)/(a+b) | EBGM05>2 |
|  | 95%CI=e^ln(EBGM)±1.96(1/a+1/b+1/c+1/d)^0.5^ |  |

Abbreviation: a, number of reports containing both the target drug and target adverse drug reaction; b, number of reports containing other adverse drug reaction of the target drug; c, number of reports containing the target adverse drug reaction of other drugs; d, number of reports containing other drugs and other adverse drug reactions. 95%CI, 95% confidence interval; N, the number of reports; χ2, chi-squared; IC, information component; IC025, the lower limit of 95% CI of the IC; E(IC), the IC expectations; V(IC), the variance of IC; EBGM, empirical Bayesian geometric mean; EBGM05, the lower limit of 95% CI of EBGM.

Supplementary Table 3 :

All adverse events meeting the positive signal threshold at the PT level from FAERS data

| PT | Case numbers | ROR(95%CI) | PRR(χ^2^) | EBGM(EBGM05) | IC(IC025) |
| --- | --- | --- | --- | --- | --- |
| Drug ineffective | 97 | 1.67 ( 1.36 - 2.06 ) | 1.64 ( 24.49 ) | 1.63 ( 1.37 ) | 0.7 ( -0.96 ) |
| Erythema | 37 | 1.65 ( 1.19 - 2.29 ) | 1.63 ( 9.06 ) | 1.62 ( 1.23 ) | 0.7 ( -0.97 ) |
| Headache | 32 | 2.69 ( 1.89 - 3.83 ) | 2.66 ( 32.52 ) | 2.62 ( 1.95 ) | 1.39 ( -0.28 ) |
| Incorrect dose administered | 32 | 1.69 ( 1.19 - 2.41 ) | 1.68 ( 8.8 ) | 1.67 ( 1.24 ) | 0.74 ( -0.93 ) |
| Fatigue | 29 | 2.7 ( 1.86 - 3.91 ) | 2.67 ( 29.69 ) | 2.63 ( 1.92 ) | 1.39 ( -0.28 ) |
| Injection site erythema | 28 | 2.24 ( 1.54 - 3.27 ) | 2.22 ( 18.5 ) | 2.19 ( 1.6 ) | 1.13 ( -0.54 ) |
| Conjunctivitis | 27 | 2.38 ( 1.62 - 3.5 ) | 2.37 ( 20.89 ) | 2.33 ( 1.69 ) | 1.22 ( -0.45 ) |
| Dizziness | 25 | 3.58 ( 2.4 - 5.35 ) | 3.55 ( 44.27 ) | 3.46 ( 2.47 ) | 1.79 ( 0.12 ) |
| Injection site pruritus | 22 | 2.85 ( 1.86 - 4.36 ) | 2.82 ( 25.3 ) | 2.77 ( 1.94 ) | 1.47 ( -0.2 ) |
| Urticaria | 21 | 2.35 ( 1.52 - 3.63 ) | 2.33 ( 15.66 ) | 2.3 ( 1.6 ) | 1.2 ( -0.47 ) |
| Nausea | 18 | 2.54 ( 1.58 - 4.06 ) | 2.52 ( 16.15 ) | 2.48 ( 1.67 ) | 1.31 ( -0.36 ) |
| Therapy interrupted | 16 | 8.45 ( 5.06 - 14.11 ) | 8.39 ( 95.82 ) | 7.79 ( 5.07 ) | 2.96 ( 1.28 ) |
| Malaise | 15 | 2.79 ( 1.67 - 4.67 ) | 2.78 ( 16.63 ) | 2.73 ( 1.77 ) | 1.45 ( -0.22 ) |
| Hypersensitivity | 15 | 2.11 ( 1.26 - 3.52 ) | 2.1 ( 8.46 ) | 2.07 ( 1.35 ) | 1.05 ( -0.62 ) |
| Alopecia | 15 | 2.28 ( 1.37 - 3.82 ) | 2.27 ( 10.48 ) | 2.24 ( 1.46 ) | 1.17 ( -0.51 ) |
| Injection site rash | 15 | 2.81 ( 1.68 - 4.71 ) | 2.8 ( 16.9 ) | 2.75 ( 1.79 ) | 1.46 ( -0.21 ) |
| Eye pain | 15 | 2.95 ( 1.76 - 4.94 ) | 2.94 ( 18.63 ) | 2.88 ( 1.87 ) | 1.53 ( -0.15 ) |
| Nasopharyngitis | 14 | 1.84 ( 1.08 - 3.13 ) | 1.83 ( 5.21 ) | 1.82 ( 1.17 ) | 0.86 ( -0.81 ) |
| Syncope | 12 | 10.52 ( 5.79 - 19.13 ) | 10.46 ( 92.67 ) | 9.53 ( 5.78 ) | 3.25 ( 1.57 ) |
| Vomiting | 8 | 2.23 ( 1.1 - 4.5 ) | 2.22 ( 5.28 ) | 2.2 ( 1.22 ) | 1.14 ( -0.54 ) |
| Urinary tract infection | 8 | 2.22 ( 1.1 - 4.49 ) | 2.22 ( 5.24 ) | 2.19 ( 1.22 ) | 1.13 ( -0.54 ) |
| Eye infection | 8 | 3.7 ( 1.82 - 7.51 ) | 3.69 ( 15.12 ) | 3.59 ( 1.99 ) | 1.84 ( 0.17 ) |
| Feeling abnormal | 7 | 2.16 ( 1.02 - 4.57 ) | 2.15 ( 4.23 ) | 2.13 ( 1.14 ) | 1.09 ( -0.59 ) |
| Death | 7 | 3.6 ( 1.69 - 7.67 ) | 3.59 ( 12.62 ) | 3.5 ( 1.86 ) | 1.81 ( 0.13 ) |
| Adverse drug reaction | 6 | 3.72 ( 1.64 - 8.42 ) | 3.71 ( 11.46 ) | 3.61 ( 1.82 ) | 1.85 ( 0.17 ) |
| Contusion | 6 | 2.3 ( 1.02 - 5.17 ) | 2.29 ( 4.28 ) | 2.26 ( 1.15 ) | 1.18 ( -0.5 ) |
| Muscle spasms | 6 | 2.39 ( 1.06 - 5.39 ) | 2.39 ( 4.73 ) | 2.35 ( 1.19 ) | 1.24 ( -0.44 ) |
| Decreased appetite | 6 | 3.02 ( 1.34 - 6.81 ) | 3.01 ( 7.83 ) | 2.95 ( 1.49 ) | 1.56 ( -0.12 ) |
| Eyelid irritation | 5 | 5.72 ( 2.32 - 14.12 ) | 5.71 ( 18.34 ) | 5.44 ( 2.56 ) | 2.44 ( 0.75 ) |
| Anaphylactic reaction | 5 | 6.16 ( 2.49 - 15.24 ) | 6.15 ( 20.27 ) | 5.84 ( 2.74 ) | 2.55 ( 0.85 ) |
| Fungal infection | 5 | 3.61 ( 1.48 - 8.83 ) | 3.61 ( 9.08 ) | 3.51 ( 1.66 ) | 1.81 ( 0.13 ) |
| Conjunctivitis allergic | 5 | 12.99 ( 5.1 - 33.1 ) | 12.96 ( 48.63 ) | 11.54 ( 5.28 ) | 3.53 ( 1.8 ) |
| Feeling hot | 5 | 2.58 ( 1.06 - 6.29 ) | 2.58 ( 4.71 ) | 2.54 ( 1.21 ) | 1.34 ( -0.34 ) |
| Ocular discomfort | 5 | 3.51 ( 1.44 - 8.57 ) | 3.5 ( 8.62 ) | 3.41 ( 1.62 ) | 1.77 ( 0.09 ) |
| Adverse reaction | 5 | 36.99 ( 13.17 - 103.85 ) | 36.89 ( 126.11 ) | 26.92 ( 11.35 ) | 4.75 ( 2.96 ) |
| Dry mouth | 4 | 4 ( 1.47 - 10.89 ) | 4 ( 8.63 ) | 3.88 ( 1.68 ) | 1.95 ( 0.26 ) |
| Upper respiratory tract infection | 4 | 3.37 ( 1.24 - 9.14 ) | 3.37 ( 6.43 ) | 3.29 ( 1.43 ) | 1.72 ( 0.03 ) |
| Blood pressure increased | 4 | 4.93 ( 1.8 - 13.47 ) | 4.92 ( 11.88 ) | 4.73 ( 2.04 ) | 2.24 ( 0.54 ) |
| Influenza like illness | 4 | 4.05 ( 1.49 - 11.01 ) | 4.04 ( 8.78 ) | 3.92 ( 1.69 ) | 1.97 ( 0.28 ) |
| Chest discomfort | 4 | 3.37 ( 1.24 - 9.14 ) | 3.37 ( 6.43 ) | 3.29 ( 1.43 ) | 1.72 ( 0.03 ) |
| Eczema herpeticum | 3 | 3.95 ( 1.24 - 12.53 ) | 3.94 ( 6.33 ) | 3.83 ( 1.45 ) | 1.94 ( 0.24 ) |
| Diverticulitis | 3 | 4.57 ( 1.43 - 14.58 ) | 4.57 ( 7.98 ) | 4.41 ( 1.67 ) | 2.14 ( 0.44 ) |
| Hot flush | 3 | 4.8 ( 1.5 - 15.32 ) | 4.8 ( 8.59 ) | 4.62 ( 1.75 ) | 2.21 ( 0.5 ) |
| Suicidal ideation | 3 | 7.58 ( 2.34 - 24.59 ) | 7.57 ( 15.86 ) | 7.09 ( 2.65 ) | 2.83 ( 1.1 ) |
| Injection site inflammation | 3 | 4.5 ( 1.41 - 14.34 ) | 4.5 ( 7.79 ) | 4.34 ( 1.65 ) | 2.12 ( 0.41 ) |
| General physical health deterioration | 3 | 4.43 ( 1.39 - 14.12 ) | 4.43 ( 7.61 ) | 4.28 ( 1.62 ) | 2.1 ( 0.39 ) |
| Blood pressure abnormal | 3 | 5.14 ( 1.61 - 16.45 ) | 5.14 ( 9.49 ) | 4.93 ( 1.86 ) | 2.3 ( 0.59 ) |
| Skin warm | 3 | 4 ( 1.26 - 12.71 ) | 4 ( 6.47 ) | 3.88 ( 1.47 ) | 1.95 ( 0.26 ) |
| Respiratory tract infection | 3 | 5.76 ( 1.8 - 18.49 ) | 5.75 ( 11.12 ) | 5.49 ( 2.07 ) | 2.46 ( 0.74 ) |
| Disease recurrence | 3 | 13.1 ( 3.92 - 43.8 ) | 13.08 ( 29.46 ) | 11.63 ( 4.24 ) | 3.54 ( 1.78 ) |
| Localised infection | 3 | 3.31 ( 1.05 - 10.48 ) | 3.31 ( 4.67 ) | 3.23 ( 1.23 ) | 1.69 ( 0 ) |
| Hip fracture | 3 | 5.05 ( 1.58 - 16.15 ) | 5.05 ( 9.26 ) | 4.85 ( 1.83 ) | 2.28 ( 0.57 ) |
| Therapy cessation | 3 | 3.31 ( 1.05 - 10.48 ) | 3.31 ( 4.67 ) | 3.23 ( 1.23 ) | 1.69 ( 0 ) |
| Throat tightness | 3 | 7.03 ( 2.17 - 22.72 ) | 7.02 ( 14.43 ) | 6.61 ( 2.48 ) | 2.72 ( 1 ) |
| Injection related reaction | 3 | 57.64 ( 13.76 - 241.37 ) | 57.55 ( 104.19 ) | 36.34 ( 10.97 ) | 5.18 ( 3.26 ) |
| Ulcerative keratitis | 3 | 13.1 ( 3.92 - 43.8 ) | 13.08 ( 29.46 ) | 11.63 ( 4.24 ) | 3.54 ( 1.78 ) |
| Atopic keratoconjunctivitis | 3 | 57.64 ( 13.76 - 241.37 ) | 57.55 ( 104.19 ) | 36.34 ( 10.97 ) | 5.18 ( 3.26 ) |
| Skin mass | 3 | 4.8 ( 1.5 - 15.32 ) | 4.8 ( 8.59 ) | 4.62 ( 1.75 ) | 2.21 ( 0.5 ) |

Abbreviation: ROR, reporting odds ratio; PRR, proportional reporting ratio; EBGM, empirical Bayesian geometric mean; EBGM05, the lower limit of the 95% CI of EBGM; IC, information component; IC025, the lower limit of the 95% CI of the IC; CI, confidence interval; PT,preferred term.

Supplementary Table 4:

Top 50 most frequent adverse events for tralokinumab at the preferred term (PT) level in males from FAERS data

| PT | Case numbers | ROR(95%CI) | PRR(χ^2^) | EBGM(EBGM05) | IC(IC025) |
| --- | --- | --- | --- | --- | --- |
| Pruritus | 49 | 1.19 ( 0.89 - 1.59 ) | 1.18 ( 1.39 ) | 1.18 ( 0.92 ) | 0.23 ( -1.43 ) |
| Drug ineffective* | 42 | 1.53 ( 1.12 - 2.09 ) | 1.5 ( 7.09 ) | 1.49 ( 1.15 ) | 0.57 ( -1.1 ) |
| Dermatitis atopic | 23 | 0.55 ( 0.36 - 0.84 ) | 0.57 ( 8.06 ) | 0.57 ( 0.4 ) | -0.82 ( -2.49 ) |
| Dry skin | 19 | 0.97 ( 0.61 - 1.53 ) | 0.97 ( 0.02 ) | 0.97 ( 0.66 ) | -0.05 ( -1.72 ) |
| Erythema* | 17 | 1.68 ( 1.04 - 2.74 ) | 1.67 ( 4.54 ) | 1.66 ( 1.1 ) | 0.73 ( -0.94 ) |
| Injection site pain | 16 | 1.15 ( 0.7 - 1.89 ) | 1.14 ( 0.29 ) | 1.14 ( 0.75 ) | 0.19 ( -1.48 ) |
| Rash | 16 | 0.51 ( 0.31 - 0.84 ) | 0.52 ( 7.15 ) | 0.53 ( 0.35 ) | -0.92 ( -2.59 ) |
| Incorrect dose administered | 14 | 1.65 ( 0.97 - 2.81 ) | 1.64 ( 3.44 ) | 1.63 ( 1.04 ) | 0.7 ( -0.97 ) |
| Dizziness* | 12 | 4.45 ( 2.48 - 7.99 ) | 4.4 ( 30.06 ) | 4.23 ( 2.59 ) | 2.08 ( 0.4 ) |
| Conjunctivitis* | 11 | 2.14 ( 1.17 - 3.91 ) | 2.13 ( 6.45 ) | 2.1 ( 1.27 ) | 1.07 ( -0.6 ) |
| Skin exfoliation | 11 | 0.91 ( 0.5 - 1.66 ) | 0.91 ( 0.09 ) | 0.91 ( 0.55 ) | -0.13 ( -1.8 ) |
| Headache* | 10 | 2.86 ( 1.51 - 5.38 ) | 2.83 ( 11.52 ) | 2.77 ( 1.63 ) | 1.47 ( -0.21 ) |
| Eczema | 10 | 0.59 ( 0.31 - 1.1 ) | 0.59 ( 2.86 ) | 0.59 ( 0.35 ) | -0.75 ( -2.42 ) |
| Fatigue* | 9 | 2.17 ( 1.11 - 4.21 ) | 2.15 ( 5.45 ) | 2.12 ( 1.22 ) | 1.09 ( -0.59 ) |
| Therapy interrupted* | 9 | 11 ( 5.48 - 22.1 ) | 10.89 ( 71.68 ) | 9.76 ( 5.44 ) | 3.29 ( 1.59 ) |
| Injection site swelling | 8 | 1.54 ( 0.76 - 3.11 ) | 1.53 ( 1.46 ) | 1.52 ( 0.85 ) | 0.61 ( -1.07 ) |
| Ocular hyperaemia | 8 | 1.05 ( 0.52 - 2.11 ) | 1.05 ( 0.02 ) | 1.04 ( 0.58 ) | 0.06 ( -1.61 ) |
| Urticaria* | 8 | 2.88 ( 1.42 - 5.84 ) | 2.86 ( 9.38 ) | 2.8 ( 1.55 ) | 1.48 ( -0.2 ) |
| Eye pain* | 8 | 4.71 ( 2.3 - 9.63 ) | 4.67 ( 21.93 ) | 4.48 ( 2.46 ) | 2.16 ( 0.48 ) |
| Dry eye | 8 | 1.06 ( 0.53 - 2.14 ) | 1.06 ( 0.03 ) | 1.06 ( 0.59 ) | 0.09 ( -1.59 ) |
| Condition aggravated | 8 | 0.93 ( 0.46 - 1.87 ) | 0.93 ( 0.04 ) | 0.93 ( 0.52 ) | -0.1 ( -1.78 ) |
| Syncope* | 7 | 14.94 ( 6.67 - 33.46 ) | 14.82 ( 76.81 ) | 12.76 ( 6.5 ) | 3.67 ( 1.95 ) |
| Eye irritation | 7 | 1.14 ( 0.54 - 2.41 ) | 1.14 ( 0.12 ) | 1.14 ( 0.61 ) | 0.19 ( -1.49 ) |
| Eye pruritus | 7 | 0.97 ( 0.46 - 2.05 ) | 0.97 ( 0.01 ) | 0.97 ( 0.52 ) | -0.05 ( -1.72 ) |
| Visual impairment* | 7 | 2.65 ( 1.24 - 5.64 ) | 2.63 ( 6.91 ) | 2.58 ( 1.37 ) | 1.37 ( -0.31 ) |
| Sleep disorder | 6 | 0.51 ( 0.23 - 1.14 ) | 0.51 ( 2.83 ) | 0.51 ( 0.26 ) | -0.96 ( -2.63 ) |
| Nasopharyngitis | 6 | 2.03 ( 0.9 - 4.58 ) | 2.02 ( 3.05 ) | 2 ( 1.01 ) | 1 ( -0.68 ) |
| Diarrhoea* | 6 | 2.62 ( 1.16 - 5.92 ) | 2.61 ( 5.77 ) | 2.56 ( 1.29 ) | 1.35 ( -0.33 ) |
| Vision blurred | 6 | 1.21 ( 0.54 - 2.71 ) | 1.21 ( 0.21 ) | 1.2 ( 0.61 ) | 0.27 ( -1.41 ) |
| Injection site erythema | 6 | 1.89 ( 0.84 - 4.25 ) | 1.88 ( 2.43 ) | 1.86 ( 0.94 ) | 0.9 ( -0.78 ) |
| Nausea* | 6 | 2.87 ( 1.27 - 6.49 ) | 2.85 ( 7.01 ) | 2.79 ( 1.41 ) | 1.48 ( -0.2 ) |
| Skin burning sensation | 5 | 2.13 ( 0.88 - 5.2 ) | 2.13 ( 2.92 ) | 2.1 ( 1 ) | 1.07 ( -0.61 ) |
| Malaise* | 5 | 2.64 ( 1.08 - 6.45 ) | 2.63 ( 4.91 ) | 2.58 ( 1.22 ) | 1.37 ( -0.32 ) |
| Vomiting* | 5 | 4.09 ( 1.66 - 10.06 ) | 4.07 ( 11.07 ) | 3.93 ( 1.85 ) | 1.97 ( 0.28 ) |
| Pain | 5 | 0.88 ( 0.36 - 2.12 ) | 0.88 ( 0.08 ) | 0.88 ( 0.42 ) | -0.19 ( -1.86 ) |
| Skin haemorrhage | 5 | 1.2 ( 0.5 - 2.92 ) | 1.2 ( 0.17 ) | 1.2 ( 0.57 ) | 0.26 ( -1.41 ) |
| Swelling face* | 5 | 5.32 ( 2.15 - 13.17 ) | 5.29 ( 16.4 ) | 5.04 ( 2.36 ) | 2.33 ( 0.64 ) |
| Urinary tract infection* | 5 | 6.08 ( 2.45 - 15.11 ) | 6.05 ( 19.68 ) | 5.71 ( 2.67 ) | 2.51 ( 0.81 ) |
| Conjunctivitis allergic* | 5 | 23.67 ( 8.76 - 63.9 ) | 23.52 ( 84.4 ) | 18.62 ( 8.11 ) | 4.22 ( 2.45 ) |
| Injection site pruritus* | 5 | 2.71 ( 1.11 - 6.61 ) | 2.7 ( 5.18 ) | 2.64 ( 1.25 ) | 1.4 ( -0.28 ) |
| Dyspnoea | 5 | 1.67 ( 0.69 - 4.06 ) | 1.67 ( 1.31 ) | 1.65 ( 0.79 ) | 0.73 ( -0.95 ) |
| Blister | 4 | 2.5 ( 0.92 - 6.77 ) | 2.49 ( 3.47 ) | 2.45 ( 1.06 ) | 1.29 ( -0.4 ) |
| Skin fissures | 4 | 0.63 ( 0.23 - 1.68 ) | 0.63 ( 0.88 ) | 0.63 ( 0.28 ) | -0.66 ( -2.34 ) |
| Lacrimation increased | 4 | 1.24 ( 0.46 - 3.34 ) | 1.24 ( 0.18 ) | 1.24 ( 0.54 ) | 0.31 ( -1.37 ) |
| Dry mouth* | 4 | 12.6 ( 4.4 - 36.11 ) | 12.54 ( 37.03 ) | 11.05 ( 4.58 ) | 3.47 ( 1.72 ) |
| Weight decreased* | 4 | 2.86 ( 1.05 - 7.75 ) | 2.85 ( 4.64 ) | 2.79 ( 1.21 ) | 1.48 ( -0.21 ) |
| Covid-19 | 4 | 0.54 ( 0.2 - 1.45 ) | 0.54 ( 1.54 ) | 0.55 ( 0.24 ) | -0.87 ( -2.54 ) |
| Rash erythematous | 4 | 1.23 ( 0.46 - 3.3 ) | 1.23 ( 0.17 ) | 1.22 ( 0.53 ) | 0.29 ( -1.39 ) |
| Injection site reaction | 4 | 1.68 ( 0.62 - 4.53 ) | 1.68 ( 1.07 ) | 1.66 ( 0.73 ) | 0.73 ( -0.95 ) |
| Ocular discomfort* | 4 | 6.94 ( 2.5 - 19.29 ) | 6.91 ( 18.71 ) | 6.47 ( 2.75 ) | 2.69 ( 0.98 ) |

Abbreviation: Asterisks (*) indicate statistically significant signals in algorithm; ROR, reporting odds ratio; PRR, proportional reporting ratio; EBGM, empirical Bayesian geometric mean; EBGM05, the lower limit of the 95% CI of EBGM; IC, information component; IC025, the lower limit of the 95% CI of the IC; CI, confidence interval; PT,preferred term; AEs, adverse events.

Supplementary Table 5:

Top 50 most frequent adverse events for tralokinumab at the PT level in females from FAERS data

| PT | Case numbers | ROR(95%CI) | PRR(χ^2^) | EBGM(EBGM05) | IC(IC025) |
| --- | --- | --- | --- | --- | --- |
| Pruritus | 58 | 1.11 ( 0.85 - 1.44 ) | 1.1 ( 0.55 ) | 1.1 ( 0.88 ) | 0.14 ( -1.53 ) |
| Drug ineffective* | 55 | 1.82 ( 1.39 - 2.39 ) | 1.78 ( 19 ) | 1.77 ( 1.4 ) | 0.82 ( -0.85 ) |
| Rash | 27 | 0.59 ( 0.4 - 0.86 ) | 0.6 ( 7.62 ) | 0.6 ( 0.43 ) | -0.74 ( -2.41 ) |
| Injection site pain | 25 | 0.91 ( 0.61 - 1.35 ) | 0.91 ( 0.22 ) | 0.91 ( 0.65 ) | -0.13 ( -1.8 ) |
| Injection site erythema | 22 | 2.47 ( 1.61 - 3.79 ) | 2.44 ( 18.48 ) | 2.41 ( 1.69 ) | 1.27 ( -0.4 ) |
| Headache* | 22 | 2.76 ( 1.8 - 4.23 ) | 2.72 ( 23.57 ) | 2.68 ( 1.87 ) | 1.42 ( -0.25 ) |
| Dermatitis atopic | 21 | 0.37 ( 0.24 - 0.57 ) | 0.38 ( 21.65 ) | 0.39 ( 0.27 ) | -1.37 ( -3.04 ) |
| Fatigue* | 20 | 3.14 ( 2 - 4.92 ) | 3.1 ( 27.79 ) | 3.04 ( 2.09 ) | 1.6 ( -0.07 ) |
| Erythema | 19 | 1.58 ( 1 - 2.49 ) | 1.57 ( 3.88 ) | 1.56 ( 1.06 ) | 0.64 ( -1.03 ) |
| Arthralgia | 19 | 1.34 ( 0.85 - 2.11 ) | 1.33 ( 1.59 ) | 1.33 ( 0.91 ) | 0.41 ( -1.26 ) |
| Incorrect dose administered* | 18 | 1.75 ( 1.09 - 2.79 ) | 1.73 ( 5.56 ) | 1.72 ( 1.16 ) | 0.78 ( -0.89 ) |
| Injection site pruritus* | 17 | 3.03 ( 1.86 - 4.93 ) | 3 ( 22.14 ) | 2.94 ( 1.96 ) | 1.56 ( -0.11 ) |
| Eye pruritus | 17 | 1.61 ( 0.99 - 2.6 ) | 1.6 ( 3.78 ) | 1.59 ( 1.06 ) | 0.67 ( -1 ) |
| Conjunctivitis* | 16 | 2.82 ( 1.71 - 4.64 ) | 2.79 ( 17.98 ) | 2.74 ( 1.8 ) | 1.46 ( -0.22 ) |
| Dry eye | 15 | 1.17 ( 0.7 - 1.95 ) | 1.17 ( 0.36 ) | 1.17 ( 0.76 ) | 0.22 ( -1.45 ) |
| Dry skin | 14 | 0.56 ( 0.33 - 0.95 ) | 0.57 ( 4.75 ) | 0.57 ( 0.36 ) | -0.82 ( -2.49 ) |
| Alopecia* | 14 | 3 ( 1.76 - 5.12 ) | 2.97 ( 17.89 ) | 2.92 ( 1.86 ) | 1.54 ( -0.13 ) |
| Urticaria* | 13 | 2.23 ( 1.28 - 3.87 ) | 2.21 ( 8.52 ) | 2.19 ( 1.38 ) | 1.13 ( -0.54 ) |
| Hypersensitivity* | 13 | 3 ( 1.72 - 5.22 ) | 2.97 ( 16.62 ) | 2.92 ( 1.83 ) | 1.55 ( -0.13 ) |
| Eczema | 12 | 0.54 ( 0.31 - 0.96 ) | 0.55 ( 4.6 ) | 0.55 ( 0.34 ) | -0.87 ( -2.54 ) |
| Injection site rash* | 12 | 3.13 ( 1.76 - 5.58 ) | 3.11 ( 16.73 ) | 3.05 ( 1.88 ) | 1.61 ( -0.07 ) |
| Skin exfoliation | 12 | 0.76 ( 0.43 - 1.34 ) | 0.76 ( 0.92 ) | 0.76 ( 0.47 ) | -0.39 ( -2.06 ) |
| Dizziness* | 12 | 2.87 ( 1.61 - 5.11 ) | 2.85 ( 14.1 ) | 2.8 ( 1.73 ) | 1.49 ( -0.19 ) |
| Condition aggravated | 11 | 0.95 ( 0.52 - 1.73 ) | 0.95 ( 0.03 ) | 0.95 ( 0.58 ) | -0.07 ( -1.74 ) |
| Injection site swelling | 11 | 1.15 ( 0.63 - 2.09 ) | 1.15 ( 0.22 ) | 1.15 ( 0.7 ) | 0.2 ( -1.47 ) |
| Nausea* | 11 | 2.34 ( 1.28 - 4.26 ) | 2.33 ( 8.17 ) | 2.3 ( 1.39 ) | 1.2 ( -0.47 ) |
| Ocular hyperaemia | 11 | 1.31 ( 0.72 - 2.38 ) | 1.31 ( 0.79 ) | 1.3 ( 0.79 ) | 0.38 ( -1.29 ) |
| Injection site reaction | 9 | 1.61 ( 0.83 - 3.11 ) | 1.6 ( 2.01 ) | 1.59 ( 0.92 ) | 0.67 ( -1 ) |
| Malaise* | 9 | 2.67 ( 1.38 - 5.19 ) | 2.66 ( 9.11 ) | 2.62 ( 1.5 ) | 1.39 ( -0.29 ) |
| Nasopharyngitis | 8 | 1.75 ( 0.87 - 3.52 ) | 1.74 ( 2.49 ) | 1.73 ( 0.96 ) | 0.79 ( -0.88 ) |
| Covid-19 | 8 | 0.69 ( 0.34 - 1.39 ) | 0.7 ( 1.07 ) | 0.7 ( 0.39 ) | -0.52 ( -2.19 ) |
| Pain | 8 | 0.93 ( 0.46 - 1.86 ) | 0.93 ( 0.05 ) | 0.93 ( 0.52 ) | -0.11 ( -1.78 ) |
| Vision blurred | 8 | 1.1 ( 0.55 - 2.22 ) | 1.1 ( 0.07 ) | 1.1 ( 0.61 ) | 0.14 ( -1.53 ) |
| Injection site bruising* | 7 | 1.55 ( 0.73 - 3.28 ) | 1.55 ( 1.35 ) | 1.54 ( 0.82 ) | 0.62 ( -1.05 ) |
| Cough | 7 | 1.59 ( 0.75 - 3.36 ) | 1.59 ( 1.5 ) | 1.58 ( 0.84 ) | 0.66 ( -1.02 ) |
| Eye pain | 7 | 2.1 ( 0.99 - 4.46 ) | 2.1 ( 3.95 ) | 2.08 ( 1.11 ) | 1.05 ( -0.62 ) |
| Injection site urticaria | 6 | 1.66 ( 0.74 - 3.72 ) | 1.65 ( 1.53 ) | 1.64 ( 0.84 ) | 0.72 ( -0.96 ) |
| Injection site mass | 6 | 2.94 ( 1.3 - 6.63 ) | 2.93 ( 7.41 ) | 2.87 ( 1.45 ) | 1.52 ( -0.16 ) |
| Feeling abnormal* | 6 | 3.29 ( 1.46 - 7.44 ) | 3.28 ( 9.24 ) | 3.21 ( 1.62 ) | 1.68 ( 0 ) |
| Skin fissures | 6 | 0.82 ( 0.37 - 1.84 ) | 0.82 ( 0.22 ) | 0.83 ( 0.42 ) | -0.28 ( -1.95 ) |
| Dyspnoea | 6 | 1.63 ( 0.72 - 3.65 ) | 1.62 ( 1.42 ) | 1.61 ( 0.82 ) | 0.69 ( -0.98 ) |
| Asthma | 6 | 1.34 ( 0.6 - 3.01 ) | 1.34 ( 0.51 ) | 1.34 ( 0.68 ) | 0.42 ( -1.26 ) |
| Therapy interrupted* | 6 | 5.56 ( 2.44 - 12.68 ) | 5.54 ( 21.2 ) | 5.31 ( 2.66 ) | 2.41 ( 0.72 ) |
| Contusion* | 5 | 2.86 ( 1.18 - 6.98 ) | 2.86 ( 5.88 ) | 2.81 ( 1.33 ) | 1.49 ( -0.19 ) |
| Asthenia* | 5 | 3.3 ( 1.35 - 8.06 ) | 3.29 ( 7.75 ) | 3.22 ( 1.53 ) | 1.69 ( 0 ) |
| Hospitalisation | 5 | 1.93 ( 0.79 - 4.67 ) | 1.92 ( 2.18 ) | 1.9 ( 0.91 ) | 0.93 ( -0.75 ) |
| Lacrimation increased | 5 | 1.41 ( 0.58 - 3.42 ) | 1.41 ( 0.59 ) | 1.4 ( 0.67 ) | 0.49 ( -1.19 ) |
| Acne* | 5 | 2.54 ( 1.04 - 6.18 ) | 2.53 ( 4.54 ) | 2.5 ( 1.19 ) | 1.32 ( -0.36 ) |
| Muscle spasms* | 5 | 3.37 ( 1.38 - 8.22 ) | 3.36 ( 8.02 ) | 3.28 ( 1.56 ) | 1.71 ( 0.03 ) |
| Rash macular | 5 | 0.93 ( 0.39 - 2.26 ) | 0.93 ( 0.02 ) | 0.94 ( 0.45 ) | -0.1 ( -1.77 ) |

Abbreviation: Asterisks (*) indicate statistically significant signals in algorithm; ROR, reporting odds ratio; PRR, proportional reporting ratio; EBGM, empirical Bayesian geometric mean; EBGM05, the lower limit of the 95% CI of EBGM; IC, information component; IC025, the lower limit of the 95% CI of the IC; CI, confidence interval; PT,preferred term; AEs, adverse events.

Supplementary Table 6:

Adverse events at the PT level for tralokinumab in patients aged under 18 from FAERS data

| PT | Case numbers | ROR(95%CI) | PRR(χ^2^) | EBGM(EBGM05) | IC(IC025) |
| --- | --- | --- | --- | --- | --- |
| Covid-19* | 1 | 26.53 ( 3.08 - 228.33 ) | 22.27 ( 20.35 ) | 22.15 ( 3.66 ) | 4.47 ( 2.47 ) |
| Product use issue* | 1 | 3.8 ( 0.44 - 32.57 ) | 3.34 ( 1.72 ) | 3.33 ( 0.55 ) | 1.74 ( -0.26 ) |
| Eye pruritus* | 1 | 21.53 ( 2.5 - 185.08 ) | 18.11 ( 16.23 ) | 18.02 ( 2.98 ) | 4.17 ( 2.17 ) |
| Injection related reaction* | 1 | 4410.2 ( 240.93 - 80729.16 ) | 3675.33 ( 1836.83 ) | 1838.17 ( 161.42 ) | 10.84 ( 8.25 ) |
| Ocular hyperaemia* | 1 | 14.96 ( 1.74 - 128.42 ) | 12.63 ( 10.82 ) | 12.59 ( 2.08 ) | 3.65 ( 1.66 ) |
| Eye swelling* | 1 | 65.63 ( 7.57 - 569.3 ) | 54.86 ( 52.26 ) | 54.06 ( 8.87 ) | 5.76 ( 3.74 ) |

Abbreviation: Asterisks (*) indicate statistically significant signals in algorithm; ROR, reporting odds ratio; PRR, proportional reporting ratio; EBGM, empirical Bayesian geometric mean; EBGM05, the lower limit of the 95% CI of EBGM; IC, information component; IC025, the lower limit of the 95% CI of the IC; CI, confidence interval; PT, preferred term.

Supplementary Table 7:

Top 50 most frequent adverse events for tralokinumab at the PT level in patients aged 18 to 65 from FAERS data

| PT | Case numbers | ROR(95%CI) | PRR(χ^2^) | EBGM(EBGM05) | IC(IC025) |
| --- | --- | --- | --- | --- | --- |
| Pruritus | 27 | 0.65 ( 0.44 - 0.96 ) | 0.67 ( 4.74 ) | 0.67 ( 0.48 ) | -0.58 ( -2.25 ) |
| Dermatitis atopic | 25 | 0.61 ( 0.41 - 0.91 ) | 0.62 ( 6.11 ) | 0.62 ( 0.45 ) | -0.68 ( -2.35 ) |
| Drug ineffective | 23 | 1.03 ( 0.68 - 1.57 ) | 1.03 ( 0.02 ) | 1.03 ( 0.73 ) | 0.04 ( -1.63 ) |
| Injection site pain | 18 | 0.98 ( 0.61 - 1.57 ) | 0.98 ( 0.01 ) | 0.98 ( 0.66 ) | -0.02 ( -1.69 ) |
| Headache* | 16 | 2.97 ( 1.8 - 4.91 ) | 2.93 ( 19.94 ) | 2.88 ( 1.89 ) | 1.53 ( -0.15 ) |
| Incorrect dose administered* | 16 | 2.75 ( 1.66 - 4.54 ) | 2.71 ( 16.96 ) | 2.67 ( 1.75 ) | 1.42 ( -0.26 ) |
| Conjunctivitis* | 15 | 3.09 ( 1.84 - 5.18 ) | 3.05 ( 20.16 ) | 2.99 ( 1.94 ) | 1.58 ( -0.09 ) |
| Dry skin | 15 | 0.73 ( 0.44 - 1.22 ) | 0.74 ( 1.44 ) | 0.74 ( 0.48 ) | -0.44 ( -2.11 ) |
| Rash | 15 | 0.47 ( 0.28 - 0.79 ) | 0.48 ( 8.61 ) | 0.49 ( 0.32 ) | -1.04 ( -2.71 ) |
| Urticaria* | 12 | 3.18 ( 1.78 - 5.67 ) | 3.15 ( 17.11 ) | 3.08 ( 1.9 ) | 1.62 ( -0.05 ) |
| Dizziness* | 11 | 3.96 ( 2.16 - 7.26 ) | 3.92 ( 23.1 ) | 3.81 ( 2.29 ) | 1.93 ( 0.25 ) |
| Injection site erythema | 11 | 1.7 ( 0.93 - 3.1 ) | 1.69 ( 3.08 ) | 1.68 ( 1.02 ) | 0.75 ( -0.92 ) |
| Skin exfoliation | 11 | 0.85 ( 0.47 - 1.55 ) | 0.85 ( 0.28 ) | 0.85 ( 0.52 ) | -0.23 ( -1.9 ) |
| Eczema | 11 | 0.65 ( 0.36 - 1.18 ) | 0.65 ( 2.03 ) | 0.66 ( 0.4 ) | -0.61 ( -2.28 ) |
| Dry eye | 11 | 1.06 ( 0.59 - 1.94 ) | 1.06 ( 0.04 ) | 1.06 ( 0.64 ) | 0.09 ( -1.58 ) |
| Hypersensitivity* | 11 | 3.95 ( 2.15 - 7.24 ) | 3.9 ( 22.97 ) | 3.8 ( 2.29 ) | 1.92 ( 0.25 ) |
| Nausea* | 10 | 3.26 ( 1.73 - 6.14 ) | 3.23 ( 15 ) | 3.16 ( 1.86 ) | 1.66 ( -0.02 ) |
| Injection site rash* | 10 | 3.97 ( 2.1 - 7.5 ) | 3.94 ( 21.16 ) | 3.83 ( 2.25 ) | 1.94 ( 0.26 ) |
| Eye pruritus | 10 | 1.18 ( 0.63 - 2.22 ) | 1.18 ( 0.28 ) | 1.18 ( 0.7 ) | 0.24 ( -1.43 ) |
| Fatigue* | 9 | 2 ( 1.03 - 3.88 ) | 1.99 ( 4.36 ) | 1.97 ( 1.13 ) | 0.98 ( -0.7 ) |
| Erythema | 9 | 0.89 ( 0.46 - 1.72 ) | 0.89 ( 0.13 ) | 0.89 ( 0.51 ) | -0.17 ( -1.84 ) |
| Arthralgia | 9 | 0.89 ( 0.46 - 1.71 ) | 0.89 ( 0.13 ) | 0.89 ( 0.51 ) | -0.17 ( -1.84 ) |
| Syncope* | 8 | 16.34 ( 7.72 - 34.58 ) | 16.19 ( 98.34 ) | 14.09 ( 7.53 ) | 3.82 ( 2.11 ) |
| Condition aggravated | 8 | 0.82 ( 0.41 - 1.65 ) | 0.82 ( 0.32 ) | 0.82 ( 0.46 ) | -0.28 ( -1.96 ) |
| Injection site pruritus | 8 | 1.87 ( 0.92 - 3.77 ) | 1.86 ( 3.12 ) | 1.84 ( 1.02 ) | 0.88 ( -0.79 ) |
| Malaise* | 7 | 3.44 ( 1.62 - 7.34 ) | 3.42 ( 11.63 ) | 3.34 ( 1.77 ) | 1.74 ( 0.06 ) |
| Vomiting* | 7 | 6.05 ( 2.81 - 13 ) | 6 ( 27.59 ) | 5.72 ( 3.01 ) | 2.52 ( 0.83 ) |
| Nasopharyngitis* | 7 | 2.56 ( 1.21 - 5.44 ) | 2.55 ( 6.44 ) | 2.51 ( 1.34 ) | 1.33 ( -0.35 ) |
| Asthma* | 7 | 2.35 ( 1.11 - 4.99 ) | 2.34 ( 5.25 ) | 2.31 ( 1.23 ) | 1.21 ( -0.47 ) |
| Injection site bruising | 7 | 2.05 ( 0.97 - 4.35 ) | 2.04 ( 3.66 ) | 2.02 ( 1.08 ) | 1.01 ( -0.66 ) |
| Illness* | 7 | 2.45 ( 1.15 - 5.19 ) | 2.43 ( 5.79 ) | 2.4 ( 1.28 ) | 1.26 ( -0.41 ) |
| Skin fissures | 7 | 1.05 ( 0.5 - 2.22 ) | 1.05 ( 0.02 ) | 1.05 ( 0.56 ) | 0.07 ( -1.6 ) |
| Pain | 6 | 0.9 ( 0.4 - 2.02 ) | 0.9 ( 0.06 ) | 0.9 ( 0.46 ) | -0.15 ( -1.82 ) |
| Diarrhoea* | 6 | 2.66 ( 1.18 - 6.01 ) | 2.65 ( 6.03 ) | 2.61 ( 1.32 ) | 1.38 ( -0.3 ) |
| Alopecia* | 6 | 2.27 ( 1.01 - 5.12 ) | 2.26 ( 4.16 ) | 2.24 ( 1.13 ) | 1.16 ( -0.52 ) |
| Ocular hyperaemia | 6 | 0.91 ( 0.4 - 2.03 ) | 0.91 ( 0.06 ) | 0.91 ( 0.46 ) | -0.14 ( -1.81 ) |
| Injection site reaction | 5 | 1.14 ( 0.47 - 2.76 ) | 1.14 ( 0.08 ) | 1.14 ( 0.54 ) | 0.19 ( -1.49 ) |
| Covid-19 | 5 | 0.62 ( 0.26 - 1.49 ) | 0.62 ( 1.18 ) | 0.62 ( 0.3 ) | -0.69 ( -2.36 ) |
| Cough | 5 | 1.67 ( 0.69 - 4.06 ) | 1.67 ( 1.33 ) | 1.66 ( 0.79 ) | 0.73 ( -0.95 ) |
| Injection site swelling | 5 | 0.68 ( 0.28 - 1.64 ) | 0.68 ( 0.75 ) | 0.68 ( 0.33 ) | -0.55 ( -2.22 ) |
| Inappropriate schedule of product administration | 5 | 0.48 ( 0.2 - 1.15 ) | 0.48 ( 2.86 ) | 0.48 ( 0.23 ) | -1.05 ( -2.73 ) |
| Therapy interrupted* | 5 | 5.71 ( 2.31 - 14.1 ) | 5.68 ( 18.29 ) | 5.43 ( 2.55 ) | 2.44 ( 0.75 ) |
| Visual impairment | 5 | 1.72 ( 0.71 - 4.17 ) | 1.71 ( 1.47 ) | 1.7 ( 0.81 ) | 0.77 ( -0.91 ) |
| Injection site mass | 4 | 2.45 ( 0.9 - 6.61 ) | 2.44 ( 3.32 ) | 2.4 ( 1.05 ) | 1.27 ( -0.42 ) |
| Anaphylactic reaction* | 4 | 16.26 ( 5.65 - 46.84 ) | 16.19 ( 49.15 ) | 14.09 ( 5.82 ) | 3.82 ( 2.07 ) |
| Conjunctivitis allergic* | 4 | 16.94 ( 5.86 - 48.94 ) | 16.86 ( 51.18 ) | 14.6 ( 6.01 ) | 3.87 ( 2.11 ) |
| Product use in unapproved indication | 4 | 0.23 ( 0.09 - 0.61 ) | 0.23 ( 10.36 ) | 0.23 ( 0.1 ) | -2.1 ( -3.77 ) |
| Injection site urticaria | 4 | 1.6 ( 0.59 - 4.3 ) | 1.59 ( 0.87 ) | 1.58 ( 0.69 ) | 0.66 ( -1.01 ) |
| Eye pain | 4 | 1.65 ( 0.61 - 4.44 ) | 1.65 ( 1 ) | 1.63 ( 0.71 ) | 0.71 ( -0.97 ) |
| Dyspnoea | 4 | 1.61 ( 0.6 - 4.33 ) | 1.61 ( 0.9 ) | 1.6 ( 0.7 ) | 0.67 ( -1 ) |

Abbreviation: Asterisks (*) indicate statistically significant signals in algorithm; ROR, reporting odds ratio; PRR, proportional reporting ratio; EBGM, empirical Bayesian geometric mean; EBGM05, the lower limit of the 95% CI of EBGM; IC, information component; IC025, the lower limit of the 95% CI of the IC; CI, confidence interval; PT, preferred term.

Supplementary Table 8:

Top 50 most frequent adverse events for tralokinumab at the PT level in patients aged over 65 from FAERS data

| PT | Case numbers | ROR(95%CI) | PRR(χ^2^) | EBGM(EBGM05) | IC(IC025) |
| --- | --- | --- | --- | --- | --- |
| Pruritus | 22 | 1.46 ( 0.94 - 2.26 ) | 1.42 ( 2.86 ) | 1.41 ( 0.98 ) | 0.5 ( -1.18 ) |
| Erythema* | 10 | 4.43 ( 2.33 - 8.44 ) | 4.31 ( 24.56 ) | 4.17 ( 2.43 ) | 2.06 ( 0.38 ) |
| Rash | 9 | 0.96 ( 0.49 - 1.87 ) | 0.96 ( 0.01 ) | 0.96 ( 0.55 ) | -0.06 ( -1.73 ) |
| Drug ineffective | 8 | 1.1 ( 0.54 - 2.24 ) | 1.1 ( 0.08 ) | 1.1 ( 0.61 ) | 0.14 ( -1.54 ) |
| Therapy interrupted* | 8 | 22.31 ( 10.29 - 48.35 ) | 21.7 ( 130.09 ) | 18.02 ( 9.43 ) | 4.17 ( 2.44 ) |
| Injection site pain | 7 | 1.23 ( 0.58 - 2.63 ) | 1.23 ( 0.3 ) | 1.23 ( 0.65 ) | 0.29 ( -1.39 ) |
| Fatigue* | 6 | 2.84 ( 1.25 - 6.45 ) | 2.8 ( 6.81 ) | 2.75 ( 1.39 ) | 1.46 ( -0.23 ) |
| Ocular hyperaemia* | 6 | 4.14 ( 1.81 - 9.44 ) | 4.07 ( 13.42 ) | 3.95 ( 1.98 ) | 1.98 ( 0.29 ) |
| Urinary tract infection* | 6 | 4.47 ( 1.96 - 10.21 ) | 4.4 ( 15.15 ) | 4.25 ( 2.13 ) | 2.09 ( 0.4 ) |
| Incorrect dose administered* | 5 | 2.66 ( 1.08 - 6.51 ) | 2.63 ( 4.94 ) | 2.59 ( 1.22 ) | 1.37 ( -0.32 ) |
| Death* | 5 | 4.46 ( 1.81 - 11.02 ) | 4.4 ( 12.65 ) | 4.26 ( 2 ) | 2.09 ( 0.4 ) |
| Malaise* | 5 | 4.28 ( 1.73 - 10.54 ) | 4.22 ( 11.83 ) | 4.09 ( 1.92 ) | 2.03 ( 0.34 ) |
| Fall | 5 | 1.95 ( 0.8 - 4.75 ) | 1.93 ( 2.22 ) | 1.91 ( 0.91 ) | 0.94 ( -0.75 ) |
| Swelling face* | 4 | 11.3 ( 3.99 - 31.96 ) | 11.15 ( 33.31 ) | 10.14 ( 4.25 ) | 3.34 ( 1.61 ) |
| Eye pruritus | 4 | 1.8 ( 0.67 - 4.88 ) | 1.79 ( 1.39 ) | 1.78 ( 0.77 ) | 0.83 ( -0.85 ) |
| Arthralgia | 4 | 0.96 ( 0.36 - 2.59 ) | 0.96 ( 0.01 ) | 0.96 ( 0.42 ) | -0.06 ( -1.74 ) |
| Diverticulitis* | 3 | 11.69 ( 3.52 - 38.86 ) | 11.58 ( 26.03 ) | 10.49 ( 3.84 ) | 3.39 ( 1.63 ) |
| Injection site pruritus* | 3 | 3.06 ( 0.97 - 9.72 ) | 3.04 ( 4 ) | 2.98 ( 1.13 ) | 1.58 ( -0.12 ) |
| Covid-19 | 3 | 0.88 ( 0.28 - 2.75 ) | 0.88 ( 0.05 ) | 0.88 ( 0.34 ) | -0.19 ( -1.87 ) |
| Eye infection* | 3 | 10.48 ( 3.17 - 34.62 ) | 10.38 ( 23.08 ) | 9.5 ( 3.5 ) | 3.25 ( 1.5 ) |
| Dizziness | 3 | 1.66 ( 0.53 - 5.23 ) | 1.65 ( 0.77 ) | 1.64 ( 0.63 ) | 0.72 ( -0.97 ) |
| Alopecia | 3 | 2.59 ( 0.82 - 8.2 ) | 2.57 ( 2.83 ) | 2.53 ( 0.97 ) | 1.34 ( -0.35 ) |
| Nasopharyngitis | 3 | 2.42 ( 0.77 - 7.66 ) | 2.41 ( 2.42 ) | 2.38 ( 0.91 ) | 1.25 ( -0.45 ) |
| Localised infection* | 3 | 10.86 ( 3.28 - 35.93 ) | 10.75 ( 24 ) | 9.81 ( 3.6 ) | 3.29 ( 1.54 ) |
| Conjunctivitis* | 3 | 4.6 ( 1.44 - 14.72 ) | 4.56 ( 8 ) | 4.41 ( 1.67 ) | 2.14 ( 0.43 ) |
| Blister | 2 | 2.8 ( 0.68 - 11.47 ) | 2.79 ( 2.24 ) | 2.74 ( 0.84 ) | 1.45 ( -0.25 ) |
| Skin burning sensation | 2 | 2.69 ( 0.66 - 11 ) | 2.68 ( 2.05 ) | 2.63 ( 0.81 ) | 1.4 ( -0.31 ) |
| Respiratory disorder* | 2 | 67.38 ( 11.21 - 404.84 ) | 66.91 ( 77.92 ) | 40.54 ( 9.04 ) | 5.34 ( 3.29 ) |
| Knee arthroplasty* | 2 | 4.92 ( 1.19 - 20.46 ) | 4.9 ( 5.92 ) | 4.71 ( 1.43 ) | 2.24 ( 0.51 ) |
| Injection site swelling | 2 | 1.4 ( 0.34 - 5.67 ) | 1.39 ( 0.22 ) | 1.39 ( 0.43 ) | 0.47 ( -1.22 ) |
| Kidney infection* | 2 | 12.63 ( 2.89 - 55.18 ) | 12.55 ( 18.9 ) | 11.26 ( 3.28 ) | 3.49 ( 1.69 ) |
| Accident* | 2 | 11.88 ( 2.73 - 51.69 ) | 11.81 ( 17.71 ) | 10.67 ( 3.12 ) | 3.42 ( 1.62 ) |
| Cystitis* | 2 | 7.77 ( 1.83 - 32.89 ) | 7.72 ( 10.87 ) | 7.24 ( 2.16 ) | 2.86 ( 1.1 ) |
| Asthenia | 2 | 2.21 ( 0.54 - 9.03 ) | 2.21 ( 1.29 ) | 2.18 ( 0.67 ) | 1.12 ( -0.58 ) |
| Rash erythematous | 2 | 2.14 ( 0.53 - 8.74 ) | 2.14 ( 1.19 ) | 2.11 ( 0.65 ) | 1.08 ( -0.62 ) |
| Vision blurred | 2 | 0.94 ( 0.23 - 3.79 ) | 0.94 ( 0.01 ) | 0.94 ( 0.29 ) | -0.09 ( -1.78 ) |
| Headache | 2 | 1.25 ( 0.31 - 5.06 ) | 1.25 ( 0.1 ) | 1.24 ( 0.39 ) | 0.31 ( -1.37 ) |
| Skin warm* | 2 | 18.37 ( 4.05 - 83.27 ) | 18.25 ( 27.6 ) | 15.59 ( 4.4 ) | 3.96 ( 2.12 ) |
| Dry skin | 2 | 0.47 ( 0.12 - 1.88 ) | 0.47 ( 1.2 ) | 0.47 ( 0.15 ) | -1.08 ( -2.76 ) |
| Eyelid margin crusting* | 2 | 8.08 ( 1.9 - 34.28 ) | 8.03 ( 11.41 ) | 7.51 ( 2.24 ) | 2.91 ( 1.15 ) |
| Coronary artery bypass* | 2 | 40.43 ( 7.81 - 209.26 ) | 40.14 ( 54.54 ) | 28.96 ( 7.32 ) | 4.86 ( 2.9 ) |
| Viral infection* | 2 | 13.47 ( 3.07 - 59.18 ) | 13.38 ( 20.23 ) | 11.92 ( 3.46 ) | 3.58 ( 1.77 ) |
| Staphylococcal infection* | 2 | 11.22 ( 2.59 - 48.61 ) | 11.15 ( 16.65 ) | 10.14 ( 2.97 ) | 3.34 ( 1.55 ) |
| Pain in extremity | 2 | 1.22 ( 0.3 - 4.94 ) | 1.22 ( 0.08 ) | 1.21 ( 0.38 ) | 0.28 ( -1.41 ) |
| Pneumonia | 2 | 1.47 ( 0.36 - 5.96 ) | 1.47 ( 0.29 ) | 1.46 ( 0.45 ) | 0.54 ( -1.15 ) |
| Productive cough* | 2 | 18.37 ( 4.05 - 83.27 ) | 18.25 ( 27.6 ) | 15.59 ( 4.4 ) | 3.96 ( 2.12 ) |
| Hypoaesthesia | 2 | 2.43 ( 0.59 - 9.92 ) | 2.42 ( 1.63 ) | 2.38 ( 0.73 ) | 1.25 ( -0.45 ) |
| Eye disorder | 2 | 2.24 ( 0.55 - 9.14 ) | 2.23 ( 1.33 ) | 2.2 ( 0.68 ) | 1.14 ( -0.56 ) |
| Hospitalisation | 2 | 1.97 ( 0.48 - 8.04 ) | 1.97 ( 0.94 ) | 1.95 ( 0.6 ) | 0.96 ( -0.74 ) |
| Hip fracture* | 2 | 5.46 ( 1.31 - 22.75 ) | 5.42 ( 6.86 ) | 5.2 ( 1.57 ) | 2.38 ( 0.64 ) |

Abbreviation: Asterisks (*) indicate statistically significant signals in algorithm; ROR, reporting odds ratio; PRR, proportional reporting ratio; EBGM, empirical Bayesian geometric mean; EBGM05, the lower limit of the 95% CI of EBGM; IC, information component; IC025, the lower limit of the 95% CI of the IC; CI, confidence interval; PT, preferred term.

Supplementary Table 9:

Top 50 most frequent adverse events for tralokinumab excluding common medication co-usage at the PT level from FAERS data

| PT | Case numbers | ROR(95%CI) | PRR(χ^2^) | EBGM(EBGM05) | IC(IC025) |
| --- | --- | --- | --- | --- | --- |
| Pruritus | 94 | 1.09 ( 0.88 - 1.34 ) | 1.09 ( 0.66 ) | 1.08 ( 0.91 ) | 0.12 ( -1.55 ) |
| Drug ineffective* | 89 | 1.67 ( 1.35 - 2.07 ) | 1.64 ( 22.36 ) | 1.63 ( 1.36 ) | 0.7 ( -0.97 ) |
| Injection site pain | 39 | 1.01 ( 0.73 - 1.39 ) | 1.01 ( 0 ) | 1.01 ( 0.77 ) | 0.01 ( -1.66 ) |
| Rash | 38 | 0.53 ( 0.38 - 0.73 ) | 0.54 ( 15.34 ) | 0.54 ( 0.41 ) | -0.88 ( -2.55 ) |
| Dermatitis atopic | 37 | 0.4 ( 0.29 - 0.56 ) | 0.42 ( 31.58 ) | 0.42 ( 0.32 ) | -1.25 ( -2.92 ) |
| Erythema* | 32 | 1.55 ( 1.09 - 2.2 ) | 1.54 ( 5.98 ) | 1.53 ( 1.14 ) | 0.61 ( -1.06 ) |
| Headache* | 30 | 2.75 ( 1.9 - 3.96 ) | 2.72 ( 31.88 ) | 2.67 ( 1.97 ) | 1.42 ( -0.25 ) |
| Fatigue* | 28 | 2.84 ( 1.94 - 4.14 ) | 2.81 ( 31.95 ) | 2.76 ( 2.01 ) | 1.47 ( -0.2 ) |
| Conjunctivitis* | 27 | 2.6 ( 1.77 - 3.82 ) | 2.58 ( 25.6 ) | 2.54 ( 1.84 ) | 1.34 ( -0.32 ) |
| Injection site erythema* | 27 | 2.35 ( 1.6 - 3.46 ) | 2.33 ( 20.24 ) | 2.3 ( 1.67 ) | 1.2 ( -0.47 ) |
| Incorrect dose administered* | 26 | 1.49 ( 1.01 - 2.2 ) | 1.49 ( 4.1 ) | 1.48 ( 1.07 ) | 0.56 ( -1.1 ) |
| Dry skin | 26 | 0.63 ( 0.43 - 0.93 ) | 0.64 ( 5.41 ) | 0.64 ( 0.46 ) | -0.64 ( -2.31 ) |
| Eye pruritus* | 25 | 1.53 ( 1.03 - 2.27 ) | 1.52 ( 4.42 ) | 1.51 ( 1.08 ) | 0.6 ( -1.07 ) |
| Dry eye | 22 | 1.16 ( 0.76 - 1.78 ) | 1.16 ( 0.5 ) | 1.16 ( 0.81 ) | 0.22 ( -1.45 ) |
| Dizziness* | 22 | 3.42 ( 2.23 - 5.24 ) | 3.39 ( 35.98 ) | 3.31 ( 2.32 ) | 1.73 ( 0.06 ) |
| Injection site pruritus* | 21 | 2.96 ( 1.91 - 4.58 ) | 2.93 ( 26.16 ) | 2.88 ( 2 ) | 1.53 ( -0.14 ) |
| Urticaria* | 21 | 2.56 ( 1.66 - 3.96 ) | 2.54 ( 19.25 ) | 2.5 ( 1.74 ) | 1.32 ( -0.35 ) |
| Arthralgia | 21 | 0.98 ( 0.64 - 1.51 ) | 0.98 ( 0.01 ) | 0.98 ( 0.68 ) | -0.02 ( -1.69 ) |
| Eczema | 21 | 0.58 ( 0.38 - 0.9 ) | 0.59 ( 6.12 ) | 0.59 ( 0.41 ) | -0.76 ( -2.43 ) |
| Skin exfoliation | 20 | 0.77 ( 0.5 - 1.21 ) | 0.78 ( 1.29 ) | 0.78 ( 0.54 ) | -0.36 ( -2.03 ) |
| Ocular hyperaemia | 20 | 1.38 ( 0.88 - 2.14 ) | 1.37 ( 2 ) | 1.37 ( 0.94 ) | 0.45 ( -1.22 ) |
| Injection site swelling | 18 | 1.31 ( 0.82 - 2.09 ) | 1.31 ( 1.31 ) | 1.31 ( 0.88 ) | 0.38 ( -1.28 ) |
| Condition aggravated | 17 | 0.91 ( 0.56 - 1.46 ) | 0.91 ( 0.17 ) | 0.91 ( 0.61 ) | -0.14 ( -1.81 ) |
| Therapy interrupted* | 16 | 9.21 ( 5.52 - 15.39 ) | 9.14 ( 106.77 ) | 8.48 ( 5.52 ) | 3.08 ( 1.41 ) |
| Malaise* | 15 | 3.04 ( 1.82 - 5.1 ) | 3.03 ( 19.84 ) | 2.97 ( 1.93 ) | 1.57 ( -0.1 ) |
| Nausea* | 15 | 2.29 ( 1.37 - 3.83 ) | 2.28 ( 10.58 ) | 2.25 ( 1.46 ) | 1.17 ( -0.5 ) |
| Alopecia* | 15 | 2.49 ( 1.49 - 4.16 ) | 2.48 ( 12.95 ) | 2.44 ( 1.59 ) | 1.29 ( -0.38 ) |
| Eye pain* | 15 | 3.22 ( 1.92 - 5.39 ) | 3.2 ( 22.07 ) | 3.13 ( 2.04 ) | 1.65 ( -0.02 ) |
| Hypersensitivity* | 14 | 2.14 ( 1.26 - 3.64 ) | 2.13 ( 8.26 ) | 2.11 ( 1.35 ) | 1.08 ( -0.6 ) |
| Vision blurred | 14 | 1.24 ( 0.73 - 2.1 ) | 1.23 ( 0.62 ) | 1.23 ( 0.79 ) | 0.3 ( -1.37 ) |
| Injection site rash* | 14 | 2.86 ( 1.68 - 4.87 ) | 2.84 ( 16.31 ) | 2.79 ( 1.79 ) | 1.48 ( -0.19 ) |
| Pain | 13 | 0.98 ( 0.57 - 1.7 ) | 0.98 ( 0.01 ) | 0.98 ( 0.62 ) | -0.03 ( -1.7 ) |
| Nasopharyngitis* | 13 | 1.86 ( 1.07 - 3.22 ) | 1.85 ( 5.02 ) | 1.84 ( 1.16 ) | 0.88 ( -0.79 ) |
| Injection site reaction | 12 | 1.57 ( 0.89 - 2.79 ) | 1.57 ( 2.46 ) | 1.56 ( 0.97 ) | 0.64 ( -1.03 ) |
| Eye irritation | 11 | 0.75 ( 0.42 - 1.37 ) | 0.76 ( 0.87 ) | 0.76 ( 0.46 ) | -0.4 ( -2.07 ) |
| Covid-19 | 11 | 0.62 ( 0.34 - 1.13 ) | 0.62 ( 2.49 ) | 0.63 ( 0.38 ) | -0.67 ( -2.34 ) |
| Skin fissures | 10 | 0.8 ( 0.43 - 1.5 ) | 0.8 ( 0.48 ) | 0.8 ( 0.48 ) | -0.31 ( -1.98 ) |
| Injection site bruising | 10 | 1.68 ( 0.9 - 3.15 ) | 1.68 ( 2.72 ) | 1.67 ( 0.99 ) | 0.74 ( -0.93 ) |
| Cough | 10 | 1.44 ( 0.77 - 2.69 ) | 1.43 ( 1.3 ) | 1.43 ( 0.85 ) | 0.51 ( -1.16 ) |
| Dyspnoea | 10 | 1.62 ( 0.87 - 3.03 ) | 1.62 ( 2.33 ) | 1.61 ( 0.95 ) | 0.69 ( -0.99 ) |
| Syncope* | 9 | 8.36 ( 4.23 - 16.52 ) | 8.33 ( 53.77 ) | 7.78 ( 4.41 ) | 2.96 ( 1.27 ) |
| Lacrimation increased | 9 | 1.47 ( 0.76 - 2.85 ) | 1.47 ( 1.34 ) | 1.46 ( 0.84 ) | 0.55 ( -1.12 ) |
| Visual impairment | 9 | 1.38 ( 0.71 - 2.66 ) | 1.38 ( 0.91 ) | 1.37 ( 0.79 ) | 0.45 ( -1.22 ) |
| Illness | 9 | 1.19 ( 0.62 - 2.3 ) | 1.19 ( 0.27 ) | 1.19 ( 0.68 ) | 0.25 ( -1.42 ) |
| Inappropriate schedule of product administration | 9 | 0.38 ( 0.2 - 0.73 ) | 0.38 ( 8.96 ) | 0.39 ( 0.22 ) | -1.37 ( -3.04 ) |
| Urinary tract infection* | 8 | 2.42 ( 1.2 - 4.89 ) | 2.42 ( 6.51 ) | 2.38 ( 1.32 ) | 1.25 ( -0.42 ) |
| Eye infection* | 8 | 4.03 ( 1.99 - 8.19 ) | 4.02 ( 17.5 ) | 3.91 ( 2.16 ) | 1.97 ( 0.29 ) |
| Diarrhoea | 8 | 1.56 ( 0.78 - 3.15 ) | 1.56 ( 1.59 ) | 1.55 ( 0.86 ) | 0.63 ( -1.04 ) |
| Fall | 8 | 1.73 ( 0.86 - 3.49 ) | 1.73 ( 2.42 ) | 1.72 ( 0.96 ) | 0.78 ( -0.89 ) |
| Blister | 7 | 1.79 ( 0.85 - 3.78 ) | 1.78 ( 2.38 ) | 1.77 ( 0.95 ) | 0.82 ( -0.85 ) |

Abbreviation: Asterisks (*) indicate statistically significant signals in algorithm; ROR, reporting odds ratio; PRR, proportional reporting ratio; EBGM, empirical Bayesian geometric mean; EBGM05, the lower limit of the 95% CI of EBGM; IC, information component; IC025, the lower limit of the 95% CI of the IC; CI, confidence interval; PT, preferred term.
